# A critical role for the *Drosophila* dopamine D_1_-like receptor Dop1R2 at the onset of metamorphosis

**DOI:** 10.1186/s12861-016-0115-z

**Published:** 2016-05-16

**Authors:** Kimberly Regna, Peri T. Kurshan, Benjamin N. Harwood, Adam M. Jenkins, Chao-Qiang Lai, Marc A.T. Muskavitch, Alan S. Kopin, Isabelle Draper

**Affiliations:** Department of Biology, Boston College, Chestnut Hill, MA 02467 USA; Molecular Cardiology Research Institute, Tufts Medical Center, Boston, MA 02111 USA; Jean Mayer USDA Human Nutrition Research Center on Aging, Tufts University, Boston, MA 02111 USA; Discovery Research, Biogen Idec, Cambridge, MA 02142 USA; Present Address: Department of Biology, Stanford University, California, 94305 USA

**Keywords:** Development, Dopamine, GPCR, RNAi, Transcriptome

## Abstract

**Background:**

Insect metamorphosis relies on temporal and spatial cues that are precisely controlled. Previous studies in *Drosophila* have shown that untimely activation of genes that are essential to metamorphosis results in growth defects, developmental delay and death. Multiple factors exist that safeguard these genes against dysregulated expression. The list of identified negative regulators that play such a role in *Drosophila* development continues to expand.

**Results:**

By using RNAi transgene-induced gene silencing coupled to spatio/temporal assessment, we have unraveled an important role for the *Drosophila* dopamine 1-like receptor, Dop1R2, in development. We show that Dop1R2 knockdown leads to pre-adult lethality. In adults that escape death, abnormal wing expansion and/or melanization defects occur. Furthermore we show that salivary gland expression of this GPCR during the late larval/prepupal stage is essential for the flies to survive through adulthood. In addition to RNAi-induced effects, treatment of larvae with the high affinity D1-like receptor antagonist flupenthixol, also results in developmental arrest, and in morphological defects comparable to those seen in Dop1R2 RNAi flies. To examine the basis for pupal lethality in Dop1R2 RNAi flies, we carried out transcriptome analysis. These studies revealed up-regulation of genes that respond to ecdysone, regulate morphogenesis and/or modulate defense/immunity.

**Conclusion:**

Taken together our findings suggest a role for Dop1R2 in the repression of genes that coordinate metamorphosis. Premature release of this inhibition is not tolerated by the developing fly.

**Electronic supplementary material:**

The online version of this article (doi:10.1186/s12861-016-0115-z) contains supplementary material, which is available to authorized users.

## Background

The naturally occurring catecholamine dopamine (DA) acts as a neurotransmitter and neurohormone in the central nervous system (CNS) of vertebrates and invertebrates. DA is a precursor in the biochemical pathway for the production of melanin, and is required for invertebrate cuticle sclerotization [1–4]. Increasing evidence suggests that in insects, DA and DA receptors (DARs) are involved in the regulation of larval and pupal ecdysis, as well as in metamorphosis [5–9].

DA metabolism has been studied extensively within many phylogenetic groups. The essential steps required for dopaminergic neurotransmission (i.e., DA synthesis, release, receptor activation, and reuptake) are conserved between flies and humans. DA synthesis is controlled by the rate-limiting enzyme tyrosine hydroxylase (TH), which is encoded in *Drosophila* by the *pale* locus [1–4, 10]. TH converts tyrosine to the precursor molecule L-DOPA, which is in turn converted to DA by the enzyme DOPA decarboxylase (DDC), encoded by the *Ddc* gene [5–9, 11, 12]. TH and DDC are required for normal development in *Drosophila*. Null mutations targeting either biosynthetic enzyme result in late embryonic lethality [13, 14]. More recently, elegant studies have shown that selective depletion of TH in the nervous system is well tolerated by the developing fly, and that corresponding adults have normal lifespan, albeit display behavioral deficits (e.g., motor activity, phototaxis, aversive learning) [15].

DA exerts its function by activating G protein-coupled receptors (GPCRs). The fruit fly expresses both D_1_-like and D_2_-like DA receptors, which are distinguished based on the ability of the receptor to couple to either stimulatory Gα_s_ (D_1_-like) or inhibitory Gα_i/o_ (D_2_-like) G proteins, which in turn activate downstream signaling mechanisms [16]. The fly D_1_-like receptors include Dop1R1 (synonyms: DopR1, dDA1, dumb, Dmdop1, DA1) [17, 18] and Dop1R2 (synonyms: DopR2, DAMB, DOPR99B) [19, 20], as well as the non-canonical DopEcR (synonym: dmDopEcR) [21–23]. DopEcR has a unique in vitro pharmacological profile and can be activated either by dopamine or by the steroid hormone 20-hydroxyecdysone (20E) [22]. There is only one known *Drosophila* D2-like receptor, Dop2R (synonym: DD2R, D2R), which has also been cloned and characterized [24].

In addition to modulating a range of receptor-mediated physiologies in insects [25–33], DA acts as a precursor of metabolites involved in cuticle melanization (pigmentation) [4], and is essential for the crosslinking of proteins and chitin during sclerotization (hardening) of the cuticle after eclosion [2, 34–37]. Although the importance of DA GPCRs as modifiers of adult fly behavior (including locomotor activity, memory, arousal, temperature preference, courtship, gustation, olfaction and response to drugs of abuse) is well-documented [21, 23, 25–31, 38, 39], the contribution of DA receptors in the modulation of developmental processes has remained poorly defined. DopEcR, which responds to both DA and ecdysone, has been shown to regulate sugar sensing, male courtship, and pheromone perception in adult insects [22, 23, 40]. Overexpression or a significant reduction in the expression of this receptor, however, does not compromise normal development [22]. The focus of our study is to define the role of the D_1_-like *Drosophila* DA receptor, Dop1R2, during development. This GPCR is well-conserved in arthropods, but exhibits limited homology with mammalian dopamine receptors [41, 42], suggesting a unique function for Dop1R2 that is specific to invertebrate physiology.

We have used transgenic Dop1R2 RNA interference (RNAi) *Drosophila*, and characterized the effects of Dop1R2 knockdown (KD) using the GAL4/UAS-mediated system. We demonstrate that Dop1R2 activity is critical during the third larval instar and pupal stages to ensure completion of development through adult emergence. Our investigations of the tissue/cell types that underlie the observed Dop1R2-mediated phenotypes suggest the involvement of Dop1R2 receptors expressed in the salivary glands. The Dop1R2 RNAi-induced phenotypes observed in escaper adults are recapitulated in progeny exposed to a Dop1R2 small molecule antagonist. We have identified a subset of genes that respond to Dop1R2 KD, and are essential in development. Our data provide the first indications that a peripheral dopamine receptor controls key developmental processes in *Drosophila.*

## Results

### Dop1R2 RNAi flies exhibit decreased Dop1R2 transcript levels

**Fig. 1:**
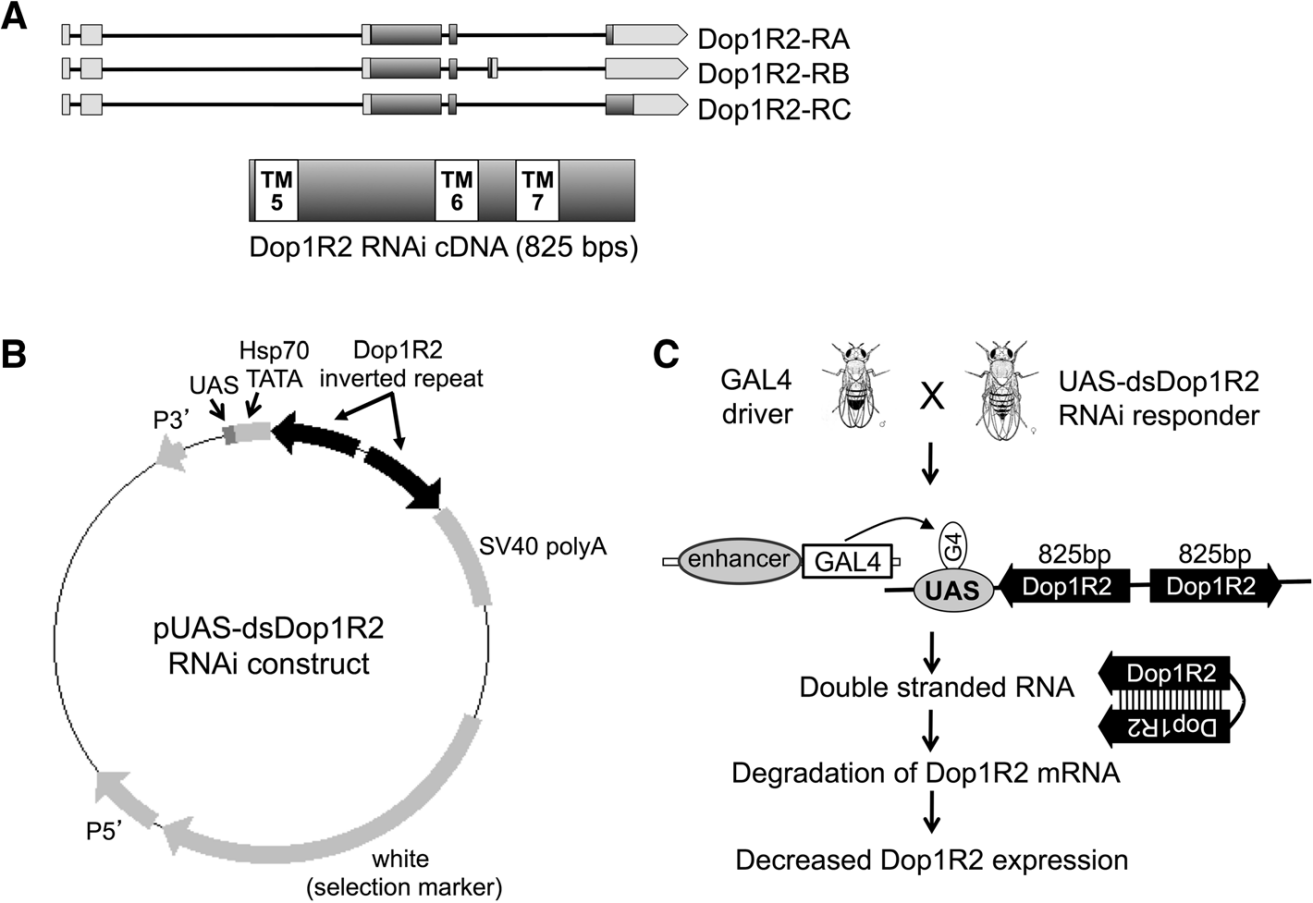
Dop1R2 cDNA and a corresponding interference construct. **a** Dop1R2 alternative transcripts Dop1R2-RA, Dop1R2-RB and Dop1R2-RC are shown. *Top*: coding sequences encompassing transmembrane domains (TMDs) 1-7 are shaded (*dark gray boxes*) and UTR regions (*light gray boxes*). *Bottom*: knockdown region expanded, with TMDs 5-7 indicated (*white boxes*). **b** pUAS-dsDop1R2 interference construct, including the yeast Upstream Activator Sequence (UAS; binding site for the yeast transcription factor, GAL4), the Dop1R2 inverted repeats and an SV40 polyadenylation site. **c** Crosses and knockdown schematic, including Dop1R2 inverted repeat (*black*)

**Fig. 2:**
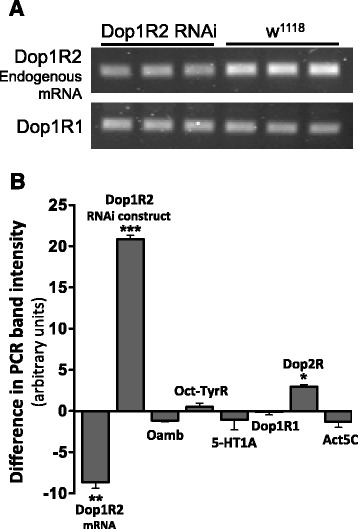
Dop1R2 RNAi flies show decreased Dop1R2 transcript levels. **a** Transcript levels assessed by RT-PCR. RNA from Dop1R2 RNAi and control flies was reverse transcribed, and PCR was performed in triplicate using primer sets corresponding to endogenous Dop1R2 as well as other biogenic amine receptors (such as Dop1R1, shown as a reference). **b** Dop1R2 transcript levels are significantly decreased in Dop1R2 RNAi flies (genotype: w^1118^;UAS-dsDop1R2/+;Act5C-GAL4/+), compared to controls (genotype: w^1118^;UAS-dsDop1R2/+;TM6B/+). The average band intensity of the Dop1R2 RNAi PCR product was compared to that of control PCR product for the same gene. Primers corresponding to Dop1R2 as well as other biogenic amine receptors (Oamb, octopamine receptor; Oct-Tyr, tyramine receptor; 5-HT1A, serotonin receptor 1A; Dop1R1, other D1-like Dopamine receptor; Dop2R, D2-like dopamine receptor) and an Actin5C control were used (Additional file 14: Table S1). The difference in PCR band intensity for the assessed genes (in Dop1R2 RNAi vs. control flies) are as follows: Dop1R2 (mRNA, in/out primers): -8.6; Dop1R2 (RNAi construct, in/in primers): 20.9; Oamb: -1.2; Oct-TyrR: 0.5; 5-HT1A: -1.1; Dop1R1: -0.1; Dop2R: 3.0; Act5C: -1.3. Symbols: Dop1R2 (Dopamine 1-like receptor 2, CG18741), Oamb (Octopamine receptor in mushroom bodies, CG3856), Oct-TyrR (Octopamine-Tyramine receptor, CG7485), 5-HT1A (5-hydroxytryptamine (serotonin) receptor 1A, CG16720), Dop1R1 (Dopamine 1-like receptor 1, CG9652), Dop2R (Dopamine 2-like receptor, CG33517), Act5C (Actin 5C, CG4027). Error bars indicated standard variance of the mean for each gene. Significance was determined for the difference in intensity of the RNAi sample PCR band versus the control w^1118^ PCR band using a one-sided *t*-test. * *p* < 0.05, ** *p* ≤ 0.01, *** *p* ≤ 0.001

Crossing UAS-dsDop1R2 RNAi transgenic flies with the Act5C-GAL4 driver strain (Fig. 1a and b) results in progeny that ubiquitously express Dop1R2 double-strand (ds) RNA (Act5C is the cytoskeletal actin 5C). This leads to targeted degradation of the endogenous Dop1R2 mRNA (i.e., Dop1R2 “knockdown”, or KD) in all tissues in which the receptor is normally expressed (Fig. 1c). When primers were designed to amplify the endogenous Dop1R2 message, without amplifying the RNAi sequence, a significant and reproducible decrease in Dop1R2 expression was observed, in Dop1R2 RNAi vs. control flies (Fig. 2a and b). When PCR primers were designed to amplify the Dop1R2 RNAi construct, a marked increase in transcript level was observed, confirming the expression of the RNAi transgene (Fig. 2b). To assess whether expression of the Dop1R2 RNAi construct could trigger off-target effects, expression of a series of other biogenic amine receptors with closest homology (36–43 % identity as assessed at the nucleotide level via ClustalW alignment [43] with Dop1R2 were also assayed. These included the second fly dopamine D_1_-like receptor Dop1R1, the dopamine D_2_-like receptor Dop2R, the octopamine receptor Oamb, the tyramine receptor Oct-TyrR and the serotonin receptor 5-HT1A. There was no significant change in the expression level of each GPCR gene under study in Dop1R2 RNAi vs. control flies, except for that of Dop2R (the D_2_-like dopamine receptor), for which a slight increase was observed (Fig. 2b).

### Expression of dsDop1R2 RNAi in the developing fly results in reduced viability, wing malformation and cuticle melanization phenotypes

**Fig. 3:**
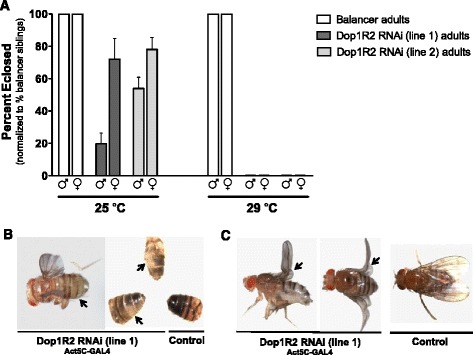
Ubiquitous knockdown of Dop1R2 results in reduced adult emergence and wing and/or melanization phenotypes. **a** Ubiquitous knockdown of Dop1R2 at 29 °C results in 100 % of Dop1R2 RNAi flies (genotype: w^1118^;UAS-dsDop1R2/+;Act5C-GAL4/+) failing to emerge, compared to control flies (genotype: w^1118^;UAS-dsDop1R2/+;TM6B/+). At 25 °C, 19.8–72.1 % (line 1) and 53.1–78.1 % (line 2) of Dop1R2 RNAi flies develop into adults (‘escapers’). 25 °C *n* = 1580 and 29 °C *n* = 449. Line 1: 4 replicates at 25 °C and 5 replicates 29 °C. Line 2: 3 replicates at 25 °C and 29 °C. Error bars indicate the Standard Error of the Mean (SEM). **b** Escaper flies may exhibit two other phenotypes: hypomelanization and curly wing. Hypomelanization phenotype appears as reduced melanization of abdominal cuticle (*arrows*). **c** Curly wing phenotype appears as bent/curved adult wing (*arrows*). Driver stock: Act5C-GAL4 (FBst0003954)

Dop1R2 RNAi flies that are reared at 29 °C and ubiquitously express the Dop1R2 RNAi construct (Additional file 1: Figure S1) develop normally throughout larval and early pupal stages, but fail to emerge from their pupal cases. When the flies are reared at a lower temperature (i.e., 25 °C), the GAL4/UAS-mediated RNAi gene silencing is attenuated [44] and ‘escaper’ adults emerge (line 1: 19.8 % males and 72.1 % females, line 2: 53.1 % males and 78.1 % females vs. control flies expressing GAL4 alone) (Fig. 3a). The escaper flies display other phenotypes with varying degrees of penetrance, including premature death, abnormal melanization (e.g., abdominal patchiness or complete absence of melanization, Fig. 3b), and/or failure to expand wings (e.g., curly wing, Fig. 3c). As with reduced viability, males show a more pronounced phenotype, with a higher penetrance, than females (data not shown). Two independent Act5C-GAL4 driver lines (FBst0004414 and FBst0003954) resulted in premature death, melanization and wing phenotypes in the progeny.

The lethal phenotype was recapitulated using two additional UAS-dsDop1R2 lines (Additional file 1: Figure S1 and Additional file 2: Figure S2) generated by the Vienna *Drosophila* RNAi Center (i.e., 3391-GD and 10524-KK, see Methods). As observed with the Draper/Kopin lab RNAi line, the male escapers (obtained with VDRC driver line 3391-GD) displayed the melanization phenotype (data not shown). Of note, in addition to the above lines, a deletion mutant of Dop1R2 (i.e., damb1) is available that removes part of the 3’coding region of the gene (personal communication, Dr. Han). The resulting transcript would encode a receptor with a truncated intracellular C-terminus. Corresponding flies are viable [45–47]. Notably, expression of a truncated GPCR in the context of a deletion that removes the last coding exons is possible, and was previously demonstrated for the *Drosophila* serotonin receptor d5-TH1A [48].

### Analysis of the temporal requirements for *Dop1R2* expression suggests a role at the third larval instar and prepupal stage

**Fig. 4:**
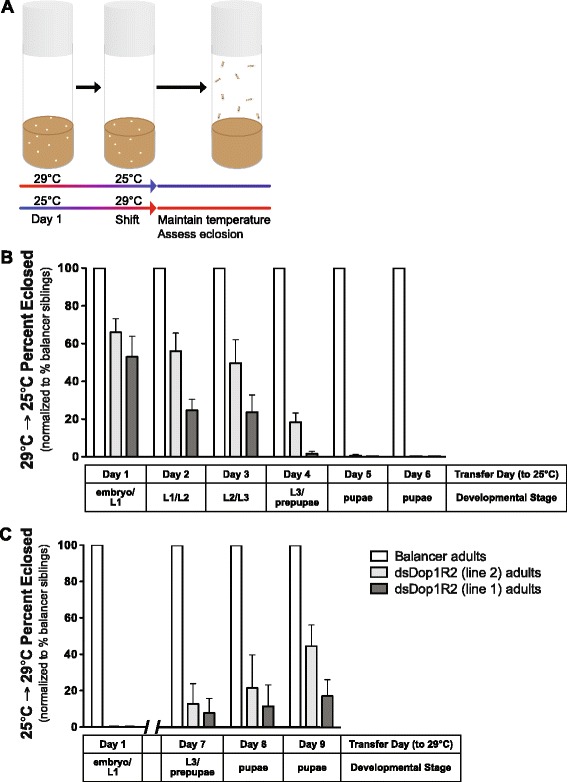
Down-regulation of Dop1R2 around larval-to-pupal ecdysis leads to developmental arrest. **a** Schematic of the temperature shift assay. **b** Analysis of progeny that were switched from 29 °C (high RNAi) to 25 °C (attenuated RNAi) on a defined day post egg laying. *n* = 1194 (line 1), *n* = 1107 (line 2). **c** Analysis of progeny that were switched from 25 °C (attenuated RNAi) to 29 °C (high RNAi) on a defined day post egg laying. *n* = 1969 (line 1), *n* = 2212 (line 2). Transfer day and temperature shift, as well as corresponding developmental stage are indicated along the x-axis. Each graph shows the percent of Dop1R2 RNAi (line 1 or line 2) (genotype: w^1118^;UAS-dsDop1R2/+;Act5C-GAL4/+) that emerge vs. controls (genotype: w^1118^;UAS-dsDop1R2/+;TM6B/+). Dop1R2 RNAi flies reared at 29 °C fail to emerge as adults. Flies transferred at 25 °C prior to the L3 larval/prepupal stage show higher eclosion. The difference in intervals between (**b**) and (**c**) reflects the temperature effect on the length of the life cycle (i.e., the life cycle is shorter when the flies primarily develop at 29 °C, and longer when the flies primarily develop at 25 °C). For each temperature shifts shown in (**b**) or (**c**), line 1 and line 2 were each assessed in triplicate. L: larval instar. Error bars indicate the Standard Error of the Mean (SEM)

We have utilized the well-established temperature effect on the GAL4/UAS system (i.e., more efficient at higher 29 °C, vs. lower 25 °C, temperature [44] to probe whether Dop1R2 expression is required during a specific time interval for the flies to complete development. Developing flies were shifted from high (29 °C) to low (25 °C) temperatures, and conversely, during different developmental stages (i.e., embryo, first/second instar larva, third feeding/wandering instar larva, early pupa, late pupa) (Fig. 4a). Regardless of which developmental stage, or direction (high to low vs. low to high), was selected to perform the transfer, flies that were kept at the high temperature throughout the third instar larval stage later arrested at the late pupal/pharate adult stage (Fig. 4b). These experiments indicate that expression of Dop1R2 at the third instar larval stage is critical for survival of the developing progeny (Fig. 4c).

### A preliminary transcriptome analysis of Dop1R2 RNAi flies reveals up-regulation of tyrosine hydroxylase and ecdysone-related genes, as well as stress and immune response genes

**Fig. 5:**
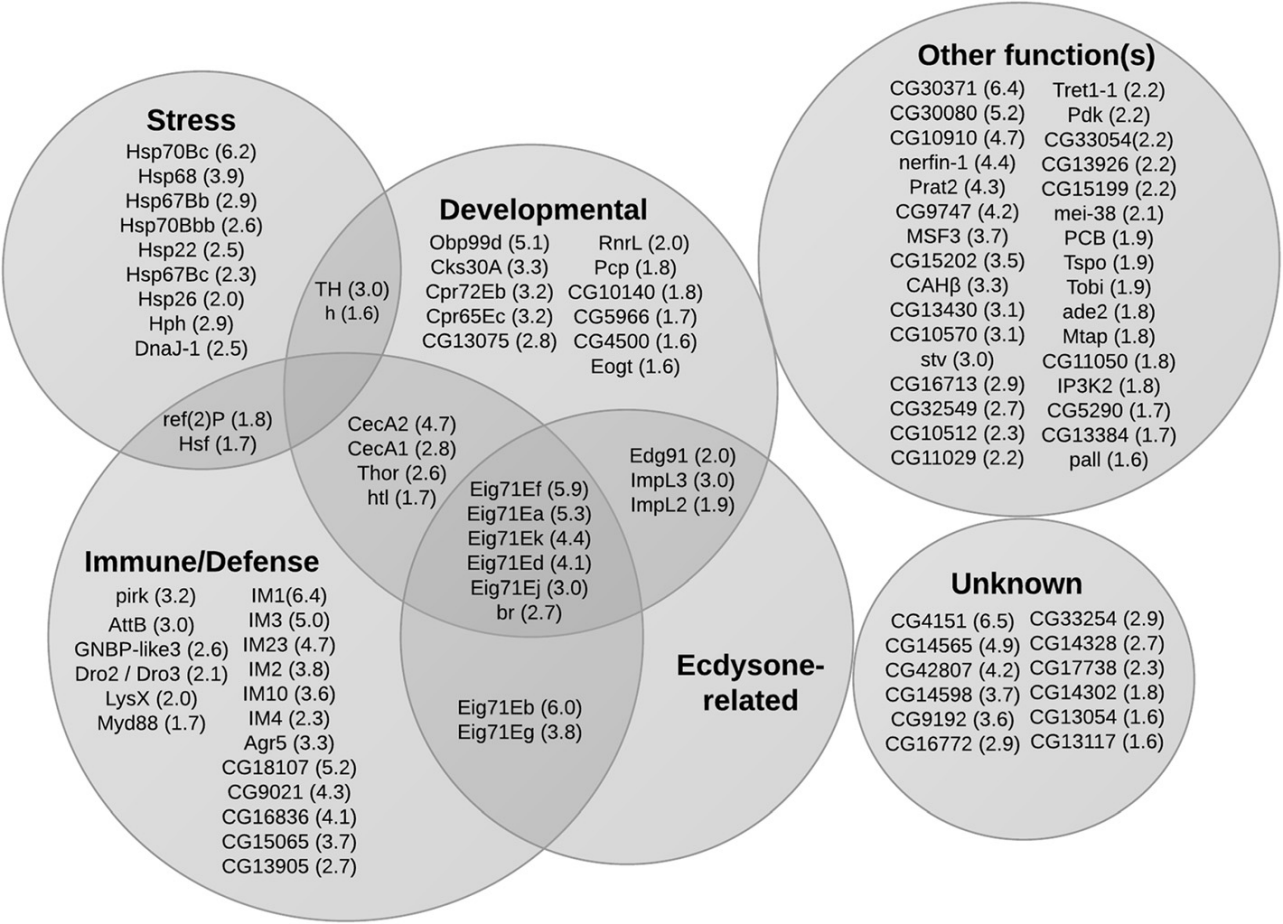
Transcriptome analysis of Dop1R2 RNAi arrested flies reveals up-regulation of families of related genes. Results indicate an increase in the expression of 101 genes that were significantly up-regulated by ≥ 1.6 times in Dop1R2 RNAi flies (genotype: w^1118^;UAS-dsDop1R2/+;Act5C-GAL4/+), compared to control flies (genotype: w^1118^;UAS-dsDop1R2/+;TM6B/+). The fold increase change in transcript level is indicated in parentheses. Statistical significance was determined using a *t*-test on the average of two independent biological replicates, with a cutoff of *p* < 0.05. Families were assigned by DAVID functional assignment and by manual annotation using FlyBase. Driver stock: Act5C-GAL4 (FBst0003954)

Affymetrix GeneChip^R^*Drosophila* genome array transcriptome expression analysis was performed in duplicate on early pupal stage Dop1R2 RNAi flies expressing the interference construct ubiquitously under restrictive conditions, and compared to that of corresponding control pupae. Significance was assessed using Genespring array analysis software (Silicon Genetics). A total of 163 genes were identified as significantly differentially expressed following assessment of the two independent transcriptome analyses (Additional file 3: Dataset 1). Among these, only eight genes were down-regulated, with a modest –1.1 to –1.5 fold-difference of expression compared to control flies. Our focus was then shifted to 101 genes that were up-regulated with a fold-difference of  ≥ 1.6 (compared to expression levels in control flies, Fig. 5 and Additional file 3: Dataset 1; arbitrary cutoff of 1.6). Results include a 3-fold increase in expression levels of tyrosine hydroxylase (TH) in Dop1R2 RNAi vs. control flies. The Affymetrix GeneChip^R^ array data discussed in this publication have been deposited into the NCBI's Gene Expression Omnibus (GEO) data repository [49] and are accessible through the GEO Series accession number GSE66496 (http://www.ncbi.nlm.nih.gov/geo/query/acc.cgi?acc=GSE66496). FlyBase annotation [50] and DAVID bioinformatic analysis [51] of the genes that are differentially expressed in response to Dop1R2 KD revealed highly significant enrichment (Benjamini corrected *p*-value range of 4.9E-2 to 4.8E-6) of genes falling under selected ontology (GO) term classes (i.e., heat shock response, immune response, salivary gland development, larval and pupal morphogenesis, Additional file 4: Dataset 2). The related genes that exhibited up-regulation include seven members in the late ecdysone-induced Eig71E (L71) gene family, which were up-regulated ~3-to 6-fold. The expression levels of multiple stress response genes (e.g., Hsp22, Hsp26, Hsp67Bb, Hsp67Bc, Hsp68, Hsp70Bbb, Hsp70Bc, Hsf), antimicrobial peptides/innate response genes (CecA1/A2, dro2/dro3, LysX, IM1, IM2, IM3, IM4, IM10, IM23) associated with gut immune responses [52], and structural components of the cuticle (Cpr72Eb, Cpr65Ec, PCP) also increased in the Dop1R2 RNAi arrested flies compared to controls (Fig. 5 and Additional file 4: Dataset 2). A parallel analysis carried out using WEB-based GEne SeT AnaLysis Toolkit (WEBGestalt) [53, 54] revealed enrichment of genes falling under similar GO term classes (Additional file 5: Figure S3). The relatedness of these groupings is further supported by the many protein-protein interactions revealed using STRING analysis (Additional file 6: Figure S4).

**Fig. 6:**
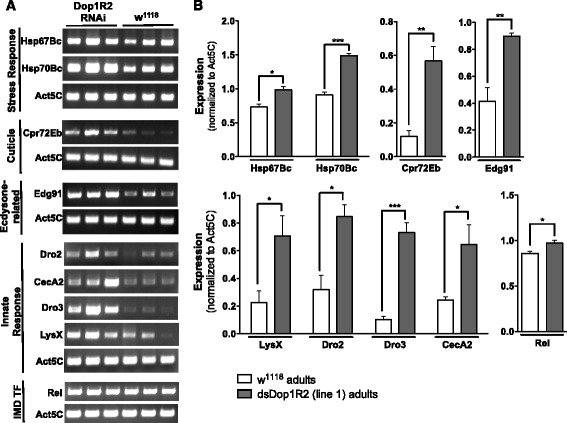
RT-PCR analysis confirms differential expression of genes in Dop1R2 RNAi flies. **a** Transcript levels assessed by RT-PCR. RNA obtained from Dop1R2 RNAi (genotype: w^1118^;UAS-dsDop1R2/+;Act5C-GAL4/+) and control pupae (genotype: w^1118^;UAS-dsDop1R2/+;TM6B/+) was reverse transcribed, and PCR was performed in triplicate using primer sets corresponding to gene of interest or to Act5C (as a normalization control). **b** Quantification of transcript levels. The average band intensity of Dop1R2 RNAi PCR products was normalized to control PCR products for Act5C. Error bars indicate the Standard Error of the Mean (SEM), and significance was determined by comparing the difference in intensities of the RNAi PCR bands versus the control PCR bands using an unpaired *t*-test. * *p* < 0.05, ** *p* ≤ 0.01, *** *p* ≤ 0.001. Driver stock: Act5C-GAL4 (FBst0003954)

To further assess results from the microarray analysis, a subset of genes was randomly picked across the main GO categories (Fig. 5). These included Hsp67Bc, Hsp70Bc (heat shock response), Cpr72Eb, Dro2, Dro3, CecA1, LysX (immune response), and Edg91 (ecdysone-dependent genes). Gene expression was assessed by RT-PCR in RNA preparations isolated from independent biological replicates (the corresponding Dop1R2 RNAi and control fly progeny were derived from three novel independent biological replicates (i.e., independent from each other, and from those used for the transcriptome analysis). This analysis confirmed increased transcript levels for all genes assessed [using RNA preparations from three independent Dop1R2 RNAi (and control) biological replicates, Fig. 6]. We also included Rel in the RT-PCR validations (although it fell below the ≥ 1.6-fold cutoff in the microarray analysis) since the corresponding protein is a key effector in the IMD pathway/gut immune response [55]. Using RT-PCR, we observed a slight, but significant, increase in Rel expression, in dsDop1R2 RNAi (vs. control) animals, as was observed by microarray analysis. Quantitative PCR confirmed an increase (4X) in TH transcript levels, in Dop1R2 RNAi flies compared to controls (Additional file 7: Figure S5).

### Analysis of the tissue-specific requirements for Dop1R2 expression suggests a role for receptor function in the salivary gland

To identify the tissue type(s) that underlie the observed phenotypes, Dop1R2 RNAi expression was directed to specific tissues/cell types utilizing a series of GAL4 drivers, and the effects of these genetic manipulations were monitored (Table 1). It is well established that Dop1R2 is abundantly expressed in the mushroom bodies (MB) [20, 56]. However, elav-mediated pan-neuronal expression of the RNAi construct (elav targets all neurons included the MB), and Tab2-mediated expression specifically targeted to the MB failed to compromise viability or to induce gross morphological abnormalities. The vast majority of drivers tested led to progeny with wild-type (WT) phenotypes (Table 1).

**Table 1:** Effect of tissue-specific down-regulation of Dop1R2. A series of GAL4 drivers was used to down-regulate Dop1R2 expression in specific tissue/cell types. The lethality observed when down-regulating expression ubiquitously was recapitulated only when using the P{GawB}332.2 driver, which expresses GAL4 in the salivary glands and amnioserosa. Semi-lethality was observed when using the P{GawB}17A and P{GawB}c729 drivers. All of the above mentioned drivers resulted in abnormal melanization and cuticle phenotypes

Gal4 driver Symbol	Expression	Phenotype
P{Act5C-GAL4}17bFO1^a,b,c^	Ubiquitous	Lethal, melanization and wing defect (in escapers)
P{Act5C-GAL4}25FO1^a,b,c,d^	Ubiquitous	Lethal, melanization and wing defect (in escapers)
P{GawB}332.3^a,b,c^	Salivary glands, amnioserosa	Lethal, melanization and wing defect (in escapers)
P{GawB}17A^a^	Salivary glands, glia, cardia	Semi-lethal
P{GawB}c729^a^	Salivary glands, female follicle cells, male accessory glands, testis sheath, cyst cells	Semi-lethal, melanization and wing defect (in escapers)
P{GawB}elav[C155]^a^	Pan-neuronal	WT
P{GawB}Tab2[201Y]^a^	Primarily in mushroom bodies	WT
P{GawB}c698a^a^	3IL CNS, not in discs	WT
P{Eip71CD-GAL4.657}TP1-1^a^	3IL brain and epidermis	WT
Bursicon-α- GAL4^a,c^	Bursicon-α positive cells	WT
P{Ccap-GAL4.P}16^a,c^	Crustacean cardioactive peptide-secreting cells	WT
P{GawB}30A^a^	Imaginal discs	WT
P{GawB}l(2)T32T32^a^	Amnioserosa, larval brain, wing discs	WT
P{GawB}c381^a^	Amnioserosa, embryonic PNS - stage 14	WT
P{Sgs3-GAL4.PD}TP1^a^	Salivary glands	WT
P{Lsp2-GAL4.H}3^a^	3IL fat body	WT
P{drm-GAL4.7.1}1.1^a^	Gastrointestinal tract, malpighian tubules	WT

All phenotypes were assessed on progeny that developed at 29 °C

^a^in combination with lab generated UAS-dsDop1R2 line 1

^b^in combination with lab generated UAS-dsDop1R2 line 2

^c^in combination with UAS-dsDop1R2 VDRC stock FBst0460369

^d^in combination with UAS-dsDop1R2 VDRC stock FBst0477151

*WT* wild-type phenotype

*3IL* third instar larva

**Fig. 7:**
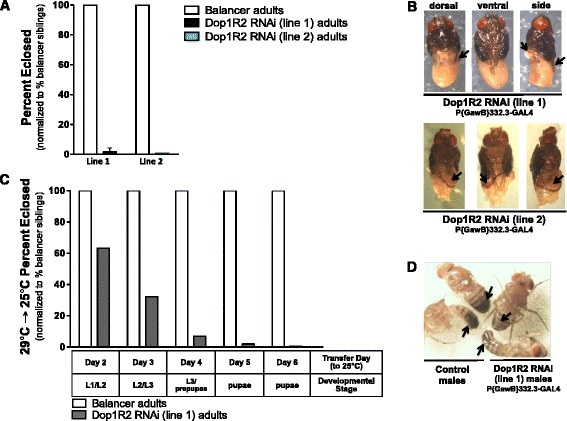
Dop1R2 down-regulation using the P{GawB}332.3 driver leads to developmental arrest at the pharate adult stage. Flies that escape lethality display a melanization phenotype. **a** Expression of Dop1R2 RNAi construct under the control of the P{GawB}332.3 driver (GAL4 expressed in the salivary glands and amnioserosa), induces 98.4 % (line 1) and 99.2 % (line 2) lethality before eclosion. Survival of RNAi flies (genotype: w^1118^;UAS-dsDop1R2/P{GawB}332.3-GAL4) is expressed as percent control progeny (genotype: w^1118^;TM6B/GawB-GAL4). *n* = 208 (line 1), *n* = 124 (line 2). Crosses with line 1 were done in triplicate; line 2 was used as a confirmatory single experiment. Error bars indicate the Standard Error of the Mean (SEM). **b** Images of pharate adults dissected out of the pupal case suggest a poorly formed abdomen (*arrows*, lines 1 and line 2) or incomplete cuticle formation (*arrows*, line 2). **c** Analysis of progeny that were switched from 29 °C (high RNAi) to 25 °C (attenuated RNAi) on a defined day post egg laying. Transfer day and temperature shift, as well as corresponding developmental stage are defined along the x-axis. Percent of Dop1R2 RNAi (line 1) (genotype: w^1118^;UAS-dsDop1R2/P{GawB}332.3-GAL4) that emerge vs. controls (genotype: w^1118^;TM6B/GawB-GAL4). Dop1R2 RNAi flies reared at 29 °C throughout development fail to emerge as adults, while those transferred to 25 °C prior to L3 larval/prepupal stage show higher eclosion. When flies are transferred between these two temperatures at different stages of development, the time course of lethality is revealed. *n* = 543. Driver stock: P{GawB}332.3-GAL4 (FBst0005398). **d** Reduced cuticle melanization (*arrows*) is observed in dsDop1R2 adult males that escape lethality at 25 °C. L: larval instar

As a follow-up to this initial study, a more focused selection of candidate drivers was tested, based on the results of the transcriptome analysis. Of particular interest was the Eig71 defensin-like peptides, which are highly expressed in one tissue – the salivary gland – during the L3 wandering/white prepupal stage [57]. The P{GawB}332.3 line (FBst0005398), which expresses GAL4 in the salivary glands, was obtained and utilized to generate salivary gland-expressing Dop1R2 RNAi flies. P{GawB}332.3-directed Dop1R2 knockdown resulted in developmental arrest of the progeny at the pupal/pharate adult stage (Fig. 7a and c), as seen with ubiquitous knockdown of Dop1R2 (Fig. 4b and c). The P{GawB}332.3 knockdown flies also exhibited poorly formed tergites and sternites, with line 1 displaying the most severe phenotype (Fig. 7b). Because P{GawB}332.3 also targets amnioserosal cells, which have a role in germ band retraction and dorsal closure in the developing embryo [58], the fraction of Dop1R2 RNAi embryos hatching into first instar larvae was assessed and compared to that of corresponding control embryos. No evidence of embryonic lethality was found in RNAi-expressing organisms (Additional file 8: Figure S6). In subsequent work, we identified two additional larval salivary gland driver lines (i.e., P{GawB}c729 – FBst0006983), which also targets glia and the proventriculus, and P{GawB}17A – FBst0008474, which also targets female follicle cells, male accessory glands, testis sheath and cyst cells) that induce semi-lethality (72.1 % and 58.2 % lethality, respectively) in the corresponding Dop1R2 RNAi progeny (Fig. 7a and Additional file 9: Figure S7B). Importantly, FBst0005398 and FBst0006983 resulted in progeny displaying wing and/or cuticle abnormalities (Fig. 7b, d and Additional file 9: Figure S7C), as was observed with ubiquitous KD of Dop1R2. For these drivers, the lethal and abnormal morphology phenotypes showed higher penetrance in male flies vs. female flies (data not shown), as was observed with ubiquitous KD of Dop1R2. As indicated in Table 1, one tissue that is common to all three phenotype-positive GAL4 drivers is the salivary gland. Follow-up experiments confirmed GAL4-driven GFP expression in the salivary glands of corresponding larvae (Additional file 10: Figure S8), while other tissues displayed background fluorescence. A fourth salivary gland driver (FBst0006870, for which GAL4 is under the control of the *sgs3* gene promoter) did not result in reduced viability (or other phenotypes) in corresponding Dop1R2 RNAi progeny (Table 1).

**Fig. 8:**
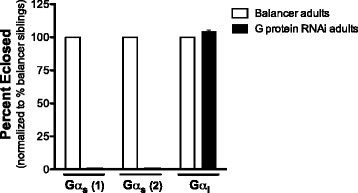
Gα_s_-targeted, but not Gα_i_-targeted KD in the salivary glands results in pre-adult lethality. Expression of either of two Gα_s_ (stimulatory G protein) RNAi constructs [122] under the control of P{GawB}332.3 driver induces lethality before eclosion (line 1 genotype: w^1118^;P{GawB}332.3-GAL4/+;UAS-dsGα_s_/+, line 2 genotype: w^1118^;P{GawB}332.3-GAL4/UAS-dsGα_s_). Expression of the Gα_i_ (inhibitory G protein) RNAi construct, using the same driver, does not compromise viability (genotype: w^1118^;P{GawB}332.3-GAL4/+;UAS-dsGα_i_/+). Survival is expressed as percent of balancer progeny. Driver stocks: Gα_s_ line 1: FBst0455666, Gα_s_ line 2: FBst0477312, Gα_i_ line: FBst0457318. Gα_s_ line 1: *n* = 141, Gα_s_ line 2: *n* = 186, Gα_i_: *n* = 416. Two replicates were performed for each of the three G protein RNAi driver lines. Error bars indicate the Standard Error of the Mean (SEM)

**Fig. 9:**
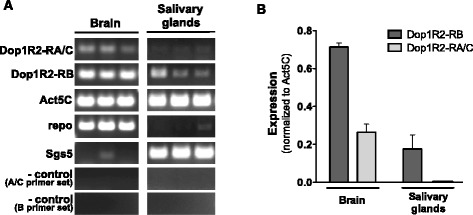
Dop1R2 is expressed in prepupal salivary glands and brain. **a** RNA obtained from the brain and salivary glands of w^1118^ prepupae was reverse transcribed, and PCR was performed in triplicate using primer sets corresponding to Dop1R2-RB, Dop1R2-RA/C or Act5C (as a normalization control). Transcript variants A/C and B are detected in the brain, and transcript variant B is detected in the salivary glands (the presence of a low abundance Dop1R2-RA/C transcript in salivary glands cannot be excluded). **b** Quantification of transcript levels (ImageJ software). Expression is quantified as band intensity for three biological replicates of Dop1R2-RB, or Dop1R2-RA/C, normalized to Act5C

Since Dop1R2 signals though the stimulatory G protein Gα_s_, we performed a complementary genetic analysis inducing G protein RNAi-mediated knockdown in vivo. Two different UAS-dsGα_s_ (stimulatory G protein) lines, as well as one UAS-dsGα_i_ (inhibitory G protein) line, were used to generate progeny at 29 °C that express dsRNA under control of the P{GawB}332.3-GAL4 driver. Crossing either Gα_s_ RNAi line with the P{GawB}332.3 resulted in pharate adult progeny that failed to eclose, as compared to the corresponding controls. However, the Gα_i_ RNAi progeny develop normally and emerge from their pupal cases as fully formed adults (Fig. 8, Table 2). These findings support the inferences that Gα_s_ signaling in the salivary glands is required for progression to the adult stage, and that the cognate GPCR(s) play an essential role in this tissue/developmental process. In contrast, the inhibitory G protein Gα_i_ does not play a critical role for development in the salivary glands. While this finding does not exclusively pinpoint Dop1R2 as the only essential Gα_s_-coupled protein in the salivary glands, it supports the premise that we are not targeting Dop2R, which signals via Gα_i_. As observed with Dop1R2 RNAi, Gα_s_ RNAi under the control of the *sgs3* promoter (FBst0006870) does not lead to compromised viability (data not shown). A follow-up molecular analysis confirmed expression of Dop1R2 in salivary glands of wild type prepupae (Fig. 9), as has been documented in other insect species (i.e., cockroach, locust, tick [59–64]).

**Table 2:** Gα_s_, but not Gα_i_, knockdown targeted to salivary glands/amnioserosa leads to developmental arrest

UAS line	Description	Phenotype
UAS-dsGα_s_ (FBst0455666)	Gα_s_ RNAi	Lethal
UAS-dsGα_s_ (FBst0477312)	Gα_s_ RNAi	Lethal
UAS-dsGα_i_ (FBst0457318)	Gα_i_ RNAi	WT

Phenotype assessed on progeny from parental Gal4 driver line P{GawB}332.3 (FBst0005398, salivary glands/amnioserosa, see Table 1) and parental UAS line, as indicated

All phenotypes were assessed on progeny that developed at 29 °C

*WT* wild-type phenotype

*ds* double-stranded

### Delivery of a Dop1R2 antagonist to larvae results in reduced viability, abnormal melanization and cuticle defects

Pharmacological assessment of Dop1R2 activity in vitro confirmed that flupenthixol dihydrochloride, with an IC_50_ of 2.6 × 10^−7^ M (Additional file 11: Figure S9), is a potent antagonist of this dopamine receptor [24, 59, 65]. Given the in vitro results, this compound was used to manipulate Dop1R2-mediated signaling in vivo, thus providing a means to complement the RNAi genetic manipulations described above. Administering flupenthixol (within a range of 0.25 mM to 4 mM) to *Drosophila* second instar larvae resulted in a dose-dependent decrease in adult eclosion with an EC_50_ of 0.8 mM (Additional file 12: Figure S10A) and developmental delay (Additional file 12: Figure S10B). When flupenthixol was administered to *Drosophila* third instar feeding larvae (at either 5 mM to 10 mM), emerging adults displayed abnormal melanization and cuticle defects (penetrance ~10–13 %, Additional file 12: Figure S10C), and these phenotypes were not observed in corresponding control flies (fed H_2_O vehicle alone). Importantly, the morphological defects resulting from administration of a Dop1R2 antagonist (as assessed in vitro) are similar to those observed with genetic knockdown of Dop1R2 (Figs. 3b, 7b and Additional file 9: Figure S7C). We cannot exclude that flupenthixol also inhibits signaling at other GPCRs [66]. However, taken together, our complementary pharmacological and genetic data support the premise that Dop1R2 regulates the observed phenotypes.

## Discussion

Our understanding of the molecular mechanisms that orchestrate the development of an adult fruit fly continues to expand. Insect metamorphosis relies on temporal and spatial cues that mediate the transition from the larval to the adult stage. Numerous gene families are tightly regulated to ensure normal insect metamorphosis, including genes that trigger larval tissue histolysis and genes that are responsible for protecting the morphing organism against microbial assault, as well as genes that mediate the formation of new adult structures. We show that a *Drosophila* dopamine receptor, i.e., the D1-like receptor Dop1R2, plays an important role in suppressing the expression of genes, which when up-regulated, lead to developmental arrest.

By using a reverse genetic approach, we show that ubiquitous knockdown of Dop1R2 results in pre-adult lethality that is dependent on receptor function during the third instar larval stage (Figs. 3 and 4). Dop1R2 RNAi adult flies that escape pre-adult lethality display multiple morphological phenotypes including hypomelanization, abnormally shaped/curly wings and defects in the cuticle (in the tergum) (Fig. 3b, c and 7b). The curly wing phenotype displayed by Dop1R2 RNAi escapers is very similar to that seen in flies that overexpress (2-fold increase) tyrosine hydroxylase (TH) in dopaminergic cells [34]. In agreement with this observation, TH is among the genes that respond to reduction in Dop1R2 knockdown (2-4 fold increase in expression levels vs. controls) (Fig. 5, Additional file 7: Figure S5, Additional file 3: Dataset 1). This finding may suggest that: (i) Dop1R2 participates in the negative regulation of TH, or (ii) compensatory mechanisms are triggered to restore normal DA-mediated signaling in the dying Dop1R2 RNAi organisms. The wing and melanization abnormalities seen in Dop1R2 RNAi escapers could be the consequence of TH dysregulation in the epidermal dopaminergic cells of the wing and cuticle. These cells have been shown to synthesize/secrete DA during molting and eclosion in *Drosophila* [16]. Under normal conditions, a peak of TH activity is detected in late L3 larvae/white prepupae [67, 68], consistent with a role for Dop1R2 during these stages of development.

Decreased Dop1R2 function leads to increased transcription of several cuticular proteins (CPs), including *Edg91* and *PCP* (Figs. 5 and 6, Additional file 3: Dataset 1). Along with ecdysone, many CPs play critical roles in puparial cuticle formation and sclerotization [69]. Proteins encoded by ecdysone-dependent genes (*Edg*) include temporally regulated CPs that are induced by increased ecdysteroid levels in the hemolymph [69, 70]. In *Drosophila*, *Edg91* responds to 20E pulses and is abundantly expressed in the epidermis during early pupal development, at the time of exocuticle synthesis [71]. ‘Pupal cuticle protein’ (PCP) is also temporally regulated by 20E, and is required for a successful third larval instar to pupal developmental transition. Notably, PCP is most tightly regulated via a small 20E titer rise around the time of head eversion [69, 72]. Dysregulated expression of CPs in Dop1R2 RNAi flies may also contribute to the observed abnormal phenotypes, specifically in the tergum (Fig. 7b).

To better assess the spatial requirements underlying Dop1R2 RNAi-induced developmental arrest in *Drosophila*, we selectively drove Dop1R2 dsRNA in various tissues/cell types (Table 1). Our microarray analysis, which showed up-regulation of salivary glands specific genes (e.g., the *Eig71E* genes), suggested involvement of this tissue in mediating Dop1R2 effects. Consistent with this observation, although most tissue-specific drivers resulted in normal progeny, targeting Dop1R2 knockdown to salivary glands (using three different GAL4 drivers, Table 1) led to arrested development/abnormal tergum in corresponding pharate adults. In addition, the corresponding progeny that escaped lethality displayed melanization and/or wing defects that were highly reminiscent of the phenotypes seen following ubiquitous Dop1R2 knockdown (Table 1, Fig. 3). A follow-up molecular analysis confirmed expression of Dop1R2 in salivary glands isolated from wild type prepupae (Fig. 9). This finding correlates with previous studies in other insect species (i.e., cockroach, locust, tick), which have demonstrated dopaminergic innervation of peripheral secretory cells in the acini, and along the ducts, of the salivary glands [60–62]. More recently, D_1_-like dopamine receptors were found in the salivary glands of adult ticks and cockroaches, where they may play a role during the feeding phase, as well as modulate salivary secretion, myoepithelial cell contraction and effects of neuropeptides [59, 62, 63].

A function for Dop1R2 in salivary glands is consistent with: (i) the observed (Dop1R2 RNAi-induced) deregulation of genes that are selectively expressed in this organ (e.g., *Eig71E* genes), and (ii) the DAVID GO clustering analysis of differentially expressed genes (Figs. 5 and 6, Additional files 3 and 4), which reveals enrichment in salivary gland biological processes. A compelling example comes from the family of *Eig71E* (aka *L71*) puff genes that are (concomitantly) induced exclusively in salivary glands, and specifically during puparium formation (they are then repressed ~12 h later) [73]. It is known that the corresponding L71 small defensin-like polypeptides are secreted from the salivary glands between the prepupal cuticle and imaginal epidermis, to help protect the metamorphosing organism against infection [73]. The *Eig71E* genes participate in the secondary response to 20E (i.e., as “late” genes), which itself depends on the expression of the early-late genes *BR-C* and *E74* [74]. *BR-C* expression is also up-regulated in Dop1R2 RNAi flies, and derepression of this gene could lead to subsequent induction of the *Eig71E* genes in Dop1R2 RNAi flies. Our studies support the premise that Dop1R2 acts upstream of selected late genes. Of note, although three out of four salivary gland drivers resulted in corresponding dsDop1R2 progeny displaying comparable phenotypes (Table 1), no phenotype was observed when using the *sgs3*-GAL4 driver. This may be due to temporal discrepancy between activation of the *sgs3* glue gene promoter and that of *Dop1R2*.

The late pupal death induced by knockdown of Dop1R2 in salivary glands is reminiscent of that observed in flies that down-regulate, in the same tissue, the low abundance ecdysone receptor minor subtype EcR-A [75]. Future studies comparing the levels of EcRs and their subcellular localization in Dop1R2 RNAi flies may prove informative.

The combination of pharmacological and RNAi (i.e., via injected dsRNA) approaches to inhibit/knockdown insect D1-like receptor signaling (in the brain) has provided a powerful means to dissect the role of these receptors in regulating motor behavior [32] or gregarious behavior [33]. Further supporting the role of Dop1R2 in development, pharmacological treatment of larvae with the established D1-like receptor antagonist flupenthixol dihydrochloride [9, 41, 76–78] results in pre-adult developmental delay/arrest (Additional file 12: Figure S10A), as well as abnormal melanization and cuticle defects in escapers (Additional file 12: Figure S10C), that recapitulate those observed by genetic manipulation of Dop1R2 expression. Importantly, flupenthixol and other selected compounds that also inhibit the mosquito AaDOP2 receptor, which is the *Aedes aegypti* ortholog of the fly Dop1R2 receptor, have emerged as promising candidate insecticides to control vector arthropods [9, 66, 79]. Our analysis, which documents drug-induced morphological abnormalities in adults that escape lethality, further highlights the potential of D1-like receptor antagonists as promising insecticides. Such anatomical defects would likely compromise survival of disease-transmitting insect vectors in the field.

Notably, analysis of genes that are differentially expressed in response to reduced levels of Dop1R2 reveals that the vast majority of them (95 %) are up-regulated (Fig. 5 and Additional file 3: Dataset 1). This observation suggests that Dop1R2 may play an important role in repressing gene expression. Functional annotation analysis of the genes for which expression increases ≥ 1.6 times, using DAVID bioinformatic resources [51], identifies enrichment in genes implicated in several biological processes for which temporal regulation is critical (Fig. 5). Several of the gene clusters fall under the GO term categories defense response, immune response, and response to heat, as well as salivary gland morphogenesis and histolysis (Additional file 4: Dataset 2).

Such deregulated activation of the immune system (in response to Dop1R2 knockdown) in the developing fly may contribute to the observed lethal phenotype. It is well-established that in *Drosophila* the balance between repression and induction of the immune defense is tightly regulated, and ensures optimal growth and size at metamorphosis [80–82]. Control of the innate immunity enables larval growth amidst the plethora of bacteria and fungi found in the natural larval feeding environment and ensures high tolerance for the larval gut commensal microbiota, which has been shown to promote development [83–85]. Conversely, deregulated immune responses can alter normal fly growth and development. Abdelsadik and Roeder (2010) have demonstrated that chronic activation of the immune system of larval salivary glands is detrimental to fly development and survival [80]. Similarly, Rynes et al. (2012) have shown that chronic inflammation of the larval gut epithelium results in developmental delay, growth retardation and lethality [86].

Recent advances in the field have unraveled an exquisite interplay of negative regulators of the immune deficiency (IMD) pathway that together adapt the immune response to the microbiome encountered by the developing fly (dietary/beneficial or pathogenic). These factors are essential to larval growth and immune homeostasis [55, 81, 86–92], and loss-of-function mutations in these negative regulators can result in larval death [86]. Our results suggest that down-regulation of Dop1R2 leads to up-regulation of multiple antimicrobial peptides (AMPs), including the cecropins CecA1 and CecA2 (Figs. 5 and 6), which are gut peptides strongly induced upon infection in an IMD/relish-dependent manner [52, 93]. In non-pathogenic conditions, these AMPs are expressed during metamorphosis [93] and are regulated by ecdysone [94]. Two other AMPs, Dro2 and Dro3, together with LysX, Hsp70Bc, Hsp67Bb and Hsp22 (also on the microarray list), comprise a small group of genes that respond to changes in fly gut microbiota [95]. LysX is a known effector of IMD response [95]. Increased expression of an entire set of *Drosophila*-specific immune-induced molecules (IMs, i.e., IM1, IM2, IM3, IM4, IM10, IM23, CG18107, CG16836 and IM2-like/CG15065) is observed in Dop1R2 RNAi animals (Fig. 5 and Additional file 3: Dataset 1). These short peptides, which are normally released into the hemolymph following septic injury, are postulated to act as chemokines [96, 97]. Importantly, IM1, IM2, IM3, IM4, IM10, IM23, along with Dro2 and AttB (Fig. 5 and Additional file 3: Dataset 1), were recently identified within a group of 14 AMPs and IMs that are markedly up-regulated in mutant *Drosophila* deficient in *activating transcription factor 3*, *atf3. Atf3* plays an essential role in larval growth, and is highly expressed in the larval gut, salivary glands and Malpighian tubules [86]. The overlap between the dysregulated gene set (and associated adverse effects on development) induced by Dop1R2 deficiency, and that induced by *atf3* deficiency, suggests an important role for Dop1R2 in the control of the immune response.

In addition to antimicrobial peptides, our study shows that the expression levels of multiple heat shock/stress genes increase in response to Dop1R2 deficiency, including the major heat-inducible proteins (Hsp70Bc, Hsp70Bbc, and Hsp68), and small heat shock proteins (Hsp22 Hsp26, Hsp67Bb and Hsp67Bc) (Fig. 5 and Additional file 3: Dataset 1). These chaperones are postulated to play a role in normal development, and under non-heat shock conditions, exhibit a peak of expression during the late L3/early pupal stages [98, 99]. Expression of small hsps is regulated by a rise in the molting hormone ecdysone [100, 101]. Hsp22, Hsp67Bb and Hsp67Bc belong to a group of four hsps that regulate morphogenesis, and buffer developmental processes from environmental assault. Interestingly, the genes that encode Hsp22, Hsp26, Hsp67Bb and Hsp67Bc all cluster within a short (~5.5Kb) genomic region at cytological location 67B on chromosome 3 L (FlyBase, [102]), consistent with possible co-regulation of their expression. High levels of Hsp70 in *Drosophila* (due to one extra copy of the gene) are sufficient to decrease organismal growth, development and survival to adulthood [103]. Up-regulation of this gene alone in developing Dop1R2 RNAi flies (Figs. 5 and 6) may thus contribute to the observed lethal phenotype that results from reduced Dop1R2 function.

A complementary DAVID GO clustering analysis [51] was used to identify previously published studies with data sets that best correlate with the set of differentially expressed genes in Dop1R2 RNAi flies. Intriguingly, the two most significant reports (i.e., PMID 16990270/Benjamini E-15 and PMID 16264191/Benjamini E-11, respectively, Additional file 4: Dataset 2) both investigate chromatin remodeling and transcriptional activity during metamorphosis [104, 105]. In both studies, the authors show that deficiency in an ecdysone-dependent transcription co-factor affects expression of a limited subset of immune-related genes. The genes identified exhibit substantial overlap with those that respond to Dop1R2 knockdown (Fig. 5, in ecdysone-related and immune diagrams). In support of a potential role of Dop1R2 in the regulation of transcription, sequence analysis (http://nls-mapper.iab.keio.ac.jp/cgi-bin/NLS_Mapper_form.cgi) reveals the presence of a bipartite nuclear localization signal (the major class of NLS found in nuclear proteins), as well as a BAF1/ABF1 chromatin reorganizing factor motif (http://www.genome.jp/tools/motif/) (Additional file 13: Figure S11), within the Dop1R2 protein. Both features are found nested in the third intracellular loop of the receptor. Interestingly, in mammals selected GPCRs (e.g., adrenergic, catecholaminergic) have been shown to localize at the nuclear membrane where they modulate gene expression [106–110]. Notably, Patel et al., have shown that the human orphan GPR158 (a Family C GPCR) harbors a bipartite NLS and translocates to the nucleus where it plays an essential role in modulating cell proliferation [111].

We postulate that under normal conditions, at the time of ecdysone-responsive early gene induction (i.e., during the L3 stage), Dop1R2 in the salivary glands participates in the co-repression of ecdysone-responsive late genes. We propose that the premature release of the Dop1R2 inhibitory effect (using RNAi approaches) translates into increased expression of the L71 defensin-like polypeptides, as well as a series of antimicrobial peptides, stress proteins/chaperones, cuticle and morphogenesis proteins in a de-synchronized manner. This misexpression could be highly detrimental to the developing fly, in agreement with a number of studies discussed above [80, 86, 103].

## Conclusions

Taken together, our analyses strongly suggest a role for Dop1R2 in the developmental control of genes at the onset of metamorphosis. Dop1R2 RNAi *Drosophila* display developmental arrest and morphological defects, as well as show dysregulated expression of genes involved in ecdysone response, morphogenesis and immunity. Tissue-specific RNAi- mediated knockdown of Dop1R2 reveals an important role for this receptor in salivary glands, during the larval-to-pupal ecdysis. In addition, pharmacological inhibition of Dop1R2 (as assessed in vitro) using a D_1_-like receptor small molecule antagonist recapitulates the abnormal phenotypes observed via genetic manipulation, and highlights the potential of drugs that target Dop1R2 as promising insecticides. Our study provides a framework to further probe the molecular mechanisms that contribute to Dop1R2-induced regulation of fly development.

## Methods

### *Drosophila* stocks and culture

Two independent UAS-dsDop1R2 homozygous RNAi stocks (lines 1 and 2) were originally generated at Tufts Medical Center, Boston, MA (Draper/Kopin Laboratory, the lethality phenotype was first documented with these lines). Each line harbors the RNAi transgene on the second chromosome where it is randomly inserted. Line 1 leads to Dop1R2 RNAi flies displaying more severe phenotypes than those obtained with line 2. Although the UAS-transgene expression is under the control of the GAL4/UAS system, subtle differences in expression may occur depending on sequences flanking the site of genomic integration. Two additional UAS-dsDop1R2 stocks (FBst0460369*: w^1118^;P{GD703}v3391 and FBst0477151: w^1118^;P{KK110947}VIE-260B) were later obtained from the Vienna *Drosophila* RNAi Center (VDRC, Vienna, Austria). Two UAS-dsGα_s_ stocks (FBst0455666: w^1118^;P{GD8547}v24958 and FBst0477312: P{KK107742}VIE-260B) and one UAS-dsGα_i_ stock (FBst0457318: w^1118^;P{GD12576}v28150/TM3) were also acquired from the VDRC. The w^1118^ stock and all of GAL4 driver fly lines (with the exception of Bursicon-α-GAL4) were obtained from the Bloomington *Drosophila* Stock Center (Indiana University, Bloomington, IN): FBst0003954: y^1^w^*^;P{Act5C-GAL4}17bFO1/TM6B, Tb^1^; FBst0004414: y^1^w^*^;P{Act5C-GAL4}25FO1/CyO, y^+^; FBst0000458: P{GawB}elav^C155^; FBst0004440: w^1118^;P{GawB}Tab2^201Y^; FBst0003739: P{GawB}c698a,w^1118^; FBst0006871: w^1118^;P{Eip71CD-GAL4.657}TP1-1; FBst0025685: y^1^w^*^;P{CCAP-GAL4.P}16; FBst0037534: w^*^;P{GawB}30A/CyO; FBst0005398: w^*^;P{GawB}332.3; FBst0008474: w*;P{GawB}17A/CyO; FBst0006983:w*;P{GawB}c729; FBst0006994: w^1118^;P{GawB}l(2)T32^T32^/CyO; FBst0003734: w^1118^;P{GawB}c381; FBst0006870: w^1118^;P{Sgs3-GAL4.PD}TP1; FBst0006357: y^1^w^1118^;P{Lsp2-GAL4.H}3; FBst0007098: w^1118^;P{drm-GAL4.7.1}1.1/TM3,Sb^1^, FBst0006874: w*;P{UAS-2xEGFP}AH2. The Bursicon-α-GAL4 stock was generously provided by Dr. W. Honegger (Vanderbilt University, Nashville, TN). All stocks were maintained at 25 °C in a 12 h light:12 h dark cycle on standard *Drosophila* medium [112]. *FBst0460369 is no longer available at VDRC; however, a corresponding RNAi line using the same RNAi target region is available: FBst0460377, w^1118^;P{GD703}v3392.

### Dop1R2 RNAi construct generation and corresponding UAS-dsDop1R2 transgenic flies

The pUAS-dsDop1R2 RNA interference (‘RNAi’) construct includes the yeast Upstream Activator Sequence (UAS; the binding site for the yeast transcription factor, GAL4) [113], inverted repeats of a 825 bp sequence corresponding to the 3’ coding region of the Dop1R2 receptor cDNA (bp 807–1631 of the Dop1R2 cDNA sequence, with bp 1 corresponding to the start of the translation initiation codon), and a SV40 polyadenylation site. Cloning of the sense and antisense cDNA repeats in the pUAST vector was performed as described previously for a Dop2R RNAi construct [25]. The pUAS-dsDop1R2 RNAi transgene construct (250–300 μg/ml) was coinjected with the P helper plasmid pΠ25.7wc (100 μg/ml) into preblastoderm w^1118^*Drosophila* embryos, according to standard protocols [114]. Multiple independent transformant lines containing the UAS-dsDop1R2 transgene were thus generated at Tufts Medical Center (Draper/Kopin lab) and maintained as homozygotes for the P[UAS-dsDop1R2] transgene. Two lines, designated UAS-dsDop1R2 line 1 and UAS-dsDop1R2 line 2, were used for study.

### Generation of Dop1R2 RNAi flies

The interference construct was expressed under the control of the well-characterized GAL4/UAS binary system [113]. UAS-dsDop1R2 homozygous flies, that were either generated in the laboratory or obtained from VDRC (i.e., 3391-GD and 10524-KK, see Methods), were crossed with each of the GAL4 driver lines listed in Table 1. Developing progeny were reared at either 29 °C or 25 °C. Isogenic progeny derived from a cross between the w^1118^ control strain and the corresponding GAL4 driver line were used as control flies for all molecular and phenotypic analyses.

### Phenotypic assessment

Viability, melanization and wing phenotype profiles of the Act5C-GAL4/UAS-dsDop1R2 RNAi progeny were assessed versus those of Act5C-GAL4/w^1118^ control progeny. To delineate the temporal requirements of Dop1R2 expression for adult eclosion/viability, developing flies were transferred from 25 °C (‘permissive’ condition) to 29 °C (‘restrictive’ condition) during different developmental stages, and emergence was monitored (as a function of developmental stage at transfer). In a complementary analysis, and to assess the spatial requirement of Dop1R2 expression for the organismal viability, Dop1R2 RNAi progeny that express the RNAi construct in specific tissues/cell types were generated at 29 °C, and characterized (the corresponding GAL4 drivers used in the crosses are listed in Table 1).

### Assessment of transcript knockdown in Dop1R2 RNAi

RT-PCR analysis was utilized to assess transcript levels in Dop1R2 RNAi flies that express the Dop1R2 RNA interference construct ubiquitously vs. control flies that express the GAL4 transcription factor alone. Analyses were carried out by standard PCR, which provided a cost-effective, yet highly sensitive approach to examine the multiple genes/transcripts under study. Focusing on Dop1R2 transcript levels, we previously observed that results obtained by standard PCR correlated with that obtained by q-PCR (data not shown). RNA was extracted from 10-20 pooled Dop1R2 RNAi early/pale pupae and corresponding control pupae. Total RNA was isolated using Trizol reagent (Invitrogen, Grand Island, NY), and purified using the RNeasy Kit with DNase treatment (Qiagen, Valencia, CA), according to the manufacturer’s recommendations. The RNA concentrations were quantified by spectrophotometry. First strand complementary DNA (cDNA) was generated from total RNA (5 ng/μl) using MultiScribe Reverse Transcriptase (Applied Biosystems, Carlsbad, CA). PCR was performed using the GeneAmp PCR core kit (Applied Biosystems, Carlsbad, CA) and the AmpliTaq Gold enzyme (Invitrogen, Grand Island, NY). Amplification was done using the GeneAmp PCR system 9700 thermocycler (Applied Biosystems, Carlsbad, CA). The conditions for PCR included: initial denaturation at 95 °C x 10 min; followed by 32 cycles of amplification: 94 °C x 30 s, 58 °C x 30 s and 72 °C x 1:30 min. The reaction was completed with a seven-minute final extension at 72 °C. The sequences of gene specific primers are provided in Additional file 14: Table S1. Dop1R2 primer pairs were designed to amplify: (i) an amplicon localized within the interference sequence (i.e., both forward and reverse primers anneal within RNAi sequence – “in/in pair”) to confirm expression of the RNAi repeats, as well as (ii) an amplicon that corresponds to a region of Dop1R2 mRNA within and outside the RNAi sequence (i.e., the forward anneals within RNAi sequence and the reverse anneal outside) – “in/out pair”) enabling assessment of endogenous Dop1R2 mRNA levels. To assess whether the RNAi construct exerted non-specific off-target effects, primer pairs corresponding to other biogenic amine receptors [i.e., Oamb (CG3856), Oct-TyrR (CG7485), 5-HT1A (CG16720), Dop1R1 (CG9652), Dop2R (CG33517)] were designed, so that the sequence with the most extensive homology (as assessed by NCBI BLAST analysis) between these transcripts and the Dop1R2 sequence was amplified for each, respectively. The primers were synthesized at the Tufts University Molecular Core (Tufts University, Boston, MA) and are listed in Additional file 14: Table S1. PCR products were run on a 1 % agarose gel with ethidium bromide, and photographed using a Multi Image Light Cabinet and camera (Alpha Innotech Corporation, San Leandro, CA). Alphaimager 2200 v5.04 imaging software (Alpha Innotech Corporation, San Leandro, CA) was used to visualize the bands and determine band intensity and saturation point. RT-PCR analysis was performed in triplicate using independent biological replicates. For each GPCR gene/transcript assessed, the difference in the PCR signal intensities in Dop1R2 RNAi and control w^1118^ flies were obtained and significance evaluated using a pooled variance *t*-test.

### Transcriptome analysis and RT-PCR validation

Gene expression analysis was performed on the GeneChip^R^*Drosophila* genome array (DrosGenome1) using Affymetrix Gene Array technology, according to standard Affymetrix protocols (http://www.affymetrix.com/support/technical/byproduct.affx?product=fly). Total early pupal RNA was isolated and purified as described in ‘*Assessment of transcript levels*’ above, and double-strand cDNA was obtained using SuperScript Double Stranded cDNA Synthesis kit (Invitrogen, Grand Island, NY). In vitro transcription and RNA labeling was performed using Enzo BioArray High Yield RNA transcript (Affymetrix, Santa Clara, CA), according to the manufacturer recommendations. Data were analyzed using the Microarray Suite program (Affymetrix, Santa Clara, CA), as well as Genespring array analysis software (Silicon Genetics). Only genes with expression signal called as “M” (marginal present) or “P” (present) in both replicates were selected for further statically analysis. A *t*-test was performed to assess the significance of differential expression between the transgenic RNAi lines and the controls. Only genes that exhibited significant differences (*p* <0.05) in expression levels compared to controls in both experiments were considered for further bioinformatic analysis using DAVID (see following ‘Bioinformatic analysis’). The complete analysis is provided in Additional file 3: Dataset 1. RT-PCR analysis was used to further assess/validate selected differentially expressed genes. Early pupae were collected, and total RNA was isolated using TRI reagent (Sigma-Aldrich, St. Louis, MO) according to manufacturer’s instructions. Synthesis of first strand cDNA was performed using 25 ng/μl total RNA and MMuLV Reverse Transcriptase (Invitrogen, Grand Island, NY). All primers were designed to span exon boundaries (except in circumstances of single-exon transcripts), to avoid gDNA amplification (primer sequences are provided in Additional file 14: Table S1). The conditions utilized for RT-PCR were: 95 °C for 2 min, followed by 30 cycles of 95 °C × 15 s, 50-55 °C × 30 s, 68 °C × 10 s, and completed with one cycle of 72 °C × 10 min. Samples were run on a 2 % agarose gel with ethidium bromide and imaged to measure band intensity using ImageJ software (NIH, Bethesda, MD). The PCR products were confirmed by sequencing (Eton Bioscience Inc., Boston, MA) the corresponding amplicon excised from the gel. For quantitative RT-PCR, SYBR Green fluorescence using the Quantitect SYBR Green kit (Qiagen, Valencia, CA) was used to quantify production of a PCR-generated cDNA fragment (primers sequences are listed in Additional file 14: Table S1). Amplification and data analysis were performed using the ABI Prism 7700 (Applied Biosystems, Carlsbad, CA). The PCR conditions utilized for RT-PCR were: 50 °C × 2 min, 95 °C × 15 min, followed by 40 cycles of 95 °C × 15 s, 64 °C × 45 s.

### Bioinformatic analysis

All of the identified differentially expressed genes were used for functional annotation analysis with the DAVID Bioinformatics Resource 6.7 [51]. Using the functional annotation tool for *Drosophila melanogaster*, a total of 101 genes that were up-regulated by ≥ 1.6-fold were analyzed for GO class and pathway associations. For any identified gene ontology (GO) term and pathway, enrichment was considered significant if the p-value observed was < 0.05 [115]. Alternatively, the set of genes was analyzed using **WEB**-based **GE**ne **S**e**T A**na**L**ysis **T**oolkit (WebGestalt), designed for functional genomic, proteomic and large-scale genetic studies. The program uses the hypergeometric test for enrichment evaluation analysis, and Benjamini-Hochberg multiple test adjustment, to assess enrichment significance [54, 116]. In addition, protein-protein interaction analysis was performed with STRING 9.1 for all of the genes up-regulated by ≥ 1.6-fold [117].

### Analysis of Dop1R2 expression in prepupal tissues

The brain and salivary glands were dissected from prepupal w^1118^*D. melanogaster*. For each tissue, total RNA was isolated, and cDNA was prepared using 50 ng/μl total RNA (as detailed in *‘RT-PCR validations’,* above). Dop1R2 was amplified using primers that span nucleotide positions 1521-1637 (isoforms A and C) or 1598-1711 (isoform B), with bp 1 corresponding to the start of the translation initiation codon. As an endogenous control, Act5C (CG4027) was amplified and used for normalization. To enable detection of tissue contamination, primers were designed to amplify *repo* (CG31240) cDNA (*repo* expression is enriched in glia) to provide a brain-specific probe [118]), and *sgs5* (CG7596) cDNA, to provide a salivary gland-specific probe. All primers were designed to span exon boundaries (sequences are listed in Additional file 14: Table S1) to avoid gDNA amplification. PCR conditions and imaging were performed as mentioned in the validation portion of *‘Transcriptome analysis and RT-PCR validation.’* To confirm that the amplicon corresponded to Dop1R2, DNA bands were excised from the gel and sequenced.

### In vitro Dop1R2 pharmacology

Luciferase assays were performed as previously described, with minor modifications [119]. HEK293 cells in 96-well plates were grown in serum-free Dulbecco’s modified eagle medium with antibiotics. After 48 h, cells were transfected using PEI (1 μg/ml) with the following constructs: the *Drosophila* Dopamine 1 receptor 2 (Dop1R2) cloned into pcDNA1.1 (4 ng/well), a CRE-LUC-HCL-PEST luciferase reporter gene (5 ng/well), and β-galactosidase-encoding plasmid (5 ng/well) as a transfection control. For agonist assays, cells were treated with the indicated concentrations of dopamine hydrochloride 24 h after transfection (Product H8502, Sigma, Natick, MA). For antagonist assays, butaclamol hydrochloride (Product D033, Sigma, Natick, MA) or flupenthixol dihydrochloride (Product 4057, Tocris Bioscience, Bristol, UK) was added to cells for 15 min prior to the addition of 1 μM dopamine. For both agonist and antagonist assays, cells were treated with compound for 4 h at 37 °C. Luciferase activity was quantified as an index of Dop1R2 signaling. Activity data were normalized relative to β-galactosidase activity as a control for transfection efficiency.

### In vivo treatment of larvae with a Dop1R2 small molecule antagonist

Adult w^1118^*D. melanogaster* were allowed to mate for 12 h at 25 °C to obtain developmentally synchronized eggs laid on *Drosophila* medium. All adults were removed, and larval development was allowed to continue for ~48 or ~72 h, to obtain L2 or L3 instar larvae. Flupenthixol dihydrochloride (Product 4057, Tocris Bioscience, Bristol, UK) was prepared as a 25 mM stock solution in dH_2_O. L3 larvae were fed flupenthixol at 5 mM or 10 mM. The drug solutions, or dH_2_O vehicle-only, were used to prepare instant fly food (Carolina Biological Supply Company, Burlington, NC) as follows: 0.5 g of fly food was placed into 25 x 95 mm polystyrene tubes (Dot Scientific Inc., Burton, MI) with 2 ml of solution (prepared in dH_2_O and 0.1 % (v/v) including Fast Green Fast Green FCF dye (Product F7258, Sigma-Aldrich, St. Louis, MO). Fifty L2 or thirty L3 instar larvae were inserted gently into tubes that were kept in a humid chamber at 25 °C during the course of the treatment.

## Ethics approval and consent to participate

The use of *Drosophila melanogaster* for research purposes does not require ethical approval.

## Consent for publication

Not applicable.

## Availability of data and material

The data sets supporting the results of this article are available in the NCBI's Gene Expression Omnibus data repository, http://www.ncbi.nlm.nih.gov/geo/query/acc.cgi?acc=GSE66496.

## Additional files

Additional file 1: Figure S1. dsDop1R2 RNAi constructs. Sequences of the three RNAi constructs utilized in the present study, and alignment on Dop1R2-RB mRNA GenBank reference sequence. The RNAi sequences include those used to generate Vienna *Drosophila* RNAi Center (VDRC) stocks 3391-GD (FBst0460369)/construct 703 and 105324-KK (FBst0477151)/construct 110947, as well as Tufts Medical Center (TMC) Dop1R2 lines (Materials and Methods). (PDF 120 kb) Additional file 2: Figure S2. dsDop1R2 knockdown-induced lethality is recapitulated with alternate RNAi constructs. (A) and (B) Ubiquitous knockdown of Dop1R2. (A) VDRC 3391-GD (genotype: w^1118^;UAS-dsDop1R2/+;Act5C-Gal4/+) results in 98 % lethality at 29 °C (*n* = 58) and 94 % lethality at 25 °C, or viability (*n* = 44). (B) VDRC 105324-KK (genotype: w^1118^;TM6B/+;UAS-dsDop1R2/+) results in 97 % lethality at 29 °C (*n* = 59), compared to control balancer siblings (genotypes: w^1118^;CyO/+;UAS-dsDop1R2/+ and w^1118^;CyO/UAS-dsDop1R2, respectively). All male escaper flies (*n* = 4) obtained when using the VDRC 3391-GD RNAi construct exhibited the hypomelanization phenotype (described in Fig. 7). (C) Salivary gland/amnioserosa targeted knockdown of Dop1R2 VDRC 3391-GD results in 68 % lethality at 29 °C in experimental flies (genotype: w^1118^;UAS-dsDop1R2/+;P{GawB}c729-GAL4/+), compared to controls (genotype: w^1118^;P{GawB}c729-Gal4/+) (*n* = 53). VDRC Dop1R2 knockdown stocks: 3391-GD (FBst0460369) and 105324-KK (FBst0477151). Driver stocks: Act5C-GAL4 (FBst0003954), P{GawB}17A-GAL4 (FBst0008474) and P{GawB}c729-GAL4 (FBst0006983). (PDF 230 kb) Additional file 3: Dataset 1. Summary of microarray data. (XLSX 61 kb) Additional file 4: Dataset 2. DAVID bioinformatic analysis of dsDop1R2 differentially expressed genes. DAVID GO clustering functional analysis reveals statistically significant (yellow) genes (with Benjamin corrected *p*-value of < 0.05) for biological process, cellular component, molecular function, pathway, and rank order of previously published studies that most correlate with the set of differentially expressed genes with fold-increase of ≥ 1.6. (XLS 179 kb) Additional file 5: Figure S3. WEB-based GEne SeT AnaLysis (WEBGestalt). Analysis of dsDop1R2 differentially expressed genes reveals enrichment in GO categories categorized by biological process, molecular function and cellular component. The top 10 GO categories that have a Benjamini corrected *p*-value of < 0.05 (red) and *p*-value > 0.05 (brown), as well as the non-enriched parents (black), are depicted. Each node provides: GO category, gene number in category and the adjusted *p*-value indicating the significance of enrichment. (PDF 207 kb) Additional file 6: Figure S4. STRING analysis reveals protein-protein interactions. Interactions indicated by connecting lines. Interactions predicted based on genomic content high throughput expression, co-expression and/or text-mining via STRING database (version 10) [117]. Legend indicates resource used in interaction prediction. (PDF 410 kb) Additional file 7: Figure S5. Tyrosine hydroxylase expression is increased in dsDop1R2 pupae. Dop1R2 knockdown pupae with the genotype w^1118^;UAS-dsDop1R2/+;Act5C-GAL4/+) exhibit increased TH transcript levels compared to controls (genotype: w^1118^;UAS-dsDop1R2/+;TM6B/+). Four-fold difference (i.e., two cycles of amplification) in TH expression is observed in Dop1R2 RNAi vs. controls using two independent biological replicates. Driver stock: Act5C-GAL4 (FBst0003954). (PDF 82 kb) Additional file 8 : Figure S6. Progression from egg to L1 instar. Dop1R2 RNAi (line 1) or w^1118^ flies were crossed with the P{GawB}332.3 driver line (GAL4 expressed in the salivary glands and amnioserosa [121]) to assess completion of embryogenesis. dsDop1R2 flies (genotype: w^1118^;UAS-dsDop1R2/P{GawB}332.3-GAL4) showed similar progression into L1 compared to controls (genotype: w^1118^;P{GawB}332.3-GAL4/+) (*n* = 50). Driver stock: P{GawB}332.3-GAL4 (FBst0005398). (PDF 26 kb) Additional file 9: Figure S7. dsDop1R2 knockdown-induced lethality is recapitulated with alternate salivary gland drivers. The wing and melanization phenotype is also observed in flies that survive to adulthood (A) Knockdown of Dop1R2 using a second larval salivary gland driver line (i.e., P{GawB}17A-GAL4; FBst0008474, which also targets glia and the proventriculus) results in semi-lethality in corresponding progeny. At 29 °C, 48.1 % of dsDop1R2 flies (genotype: w^1118^;UAS-dsDop1R2/P{GawB}17A-GAL4) emerged vs. control siblings (genotype: w^1118^;CyO/UAS-dsDop1R2). Total flies assessed, *n* = 397. (B) Knockdown of Dop1R2 using a third larval salivary gland driver line (i.e., P{GawB}c729-GAL4^;^ FBst0006983, which also targets follicle cells, accessory glands, testis sheath, cyst cells) also results in lethality. At 29 °C, 27.9 % of corresponding dsDop1R2 flies (genotype: w^1118^;UAS-dsDop1R2/P{GawB}c729-GAL4) emerged vs. adult controls (genotype: w^1118^;UAS-dsDop1R2, generated in parallel control cross). Total flies assessed, *n* = 211. In both (A) and (B) the expected dsDop1R2 progeny is 100 % of controls. (C) dsDop1R2 escapers (genotype: w^1118^;UAS-dsDop1R2/P{GawB} c729-GAL4) display cuticle and wing abnormalities (indicated by arrows in the left and center panels), or fail to fully eclose from pupal case (right panel). (PDF 824 kb) Additional file 10: Figure S8. Confirmation of salivary gland expression induced by the GAL4 drivers that were utilized. To confirm GAL4 expression in salivary glands, the UAS-GFP responder stock: w*;P{UAS-2xEGFP}AH2 (FBst0006874) was crossed with either (i) FBst0005398, (ii) FBst0008474 or (iii) FBst0006983, driver lines (see Table 1). The genotype of the corresponding progeny is (i) w^1118^;UAS-EGFP/P{GawB}332.3-GAL4, (ii) w^1118^;UAS-EGFP/P{GawB}17A-GAL4 and (iii) w^1118^;UAS-EGFP/P{GawB}c729-GAL4. The UAS-GFP responder stock was also crossed with w^1118^ to generate w^1118^;UAS-EGFP/+ controls. All three drivers tested resulted in marked GFP expression in the salivary glands. No overlapping fluorescence was detected in other tissue/cell type. Control flies showed dull (background) fluorescence only. All images, magnification: 100X, image exposure: 5 msec. (PDF 2465 kb) Additional file 11: Figure S9. Dop1R2 is stimulated by dopamine and antagonized by two known small molecules in vitro. Human Embryonic Kidney cells (HEK293 cells) were transiently co-transfected in a 96-well plate assay with plasmids encoding *Drosophila* Dop1R2 receptor and a luciferase reporter gene. (A) Increasing concentrations of dopamine activates the receptor (EC_50_ = 2.7 × 10^−7^ M). (B) Stimulation of the Dop1R2 receptor by dopamine (100 μM) is inhibited with increasing concentrations of either flupenthixol dihydrochloride (IC_50_ = 2.6 × 10^−7^ M) or butaclamol (IC_50_ = 21.6 × 10^−7^ M). Data represent the mean ± the Standard Error of the Mean (SEM) from three independent experiments, each performed in triplicate. Methods for this assay are as previously described [119]. (PDF 227 kb) Additional file 12: Figure S10. Exposure of *Drosophila melanogaster* (w^1118^) larvae to flupenthixol dihydrochloride results in increased lethality and developmental defect. (A) Assessment of adult eclosion following larval exposure to flupenthixol dihydrochloride reveals a concentration-dependent effect (EC_50_ = 0.8 mM). (B-C) Feeding flupenthixol (5 mM or 10 mM, 0.1 % fast green dye, in H_2_O) to L3 larvae (B) results in cuticle and melanization defects, in 13 % 10 % of adults, respectively. These defects are not observed in control flies (the corresponding larvae were fed 0.1 % fast green dye in H_2_O, only). (C) Images are showing two day old adults (5 days post-exposure onset). *n* = 30 larvae per concentration, three independent replicates. (PDF 459 kb) Additional file 13: Figure S11. Dop1R2 sequence motif. (A) GenomeNet motif analysis via (http://www.genome.jp/tools/motif/) reveals homology to BAF1/ABF1 chromatin reorganizing factor. (B) Sequence analysis via cNLS mapper (http://nls-mapper.iab.keio.ac.jp/cgi-bin/NLS_Mapper_form.cgi) reveals the presence of a bipartite nuclear localization signal. (C) The predicted BAF1/ABF1 chromatin reorganization factor motif (straight line) and a predicted bipartite nuclear localization signal (dotted line) are shown on the corresponding Dop1R2 protein sequence. TM: transmembrane, ECL: extracellular loop, ICL: intracellular loop, ICT: intracellular tail. (PDF 245 kb) Additional file 14: Table S1. Primer sequences. (DOC 44 kb)

## References

[1] Wright TRF, Hodgetts RB, Sherald AF (1976). The Genetics of Dopa Decarboxylase in Drosophila melanogaster. Genetics.

[2] Wright TRF (1987). The genetics of biogenic amine metabolism, sclerotization, and melanization in Drosophila melanogaster. Adv Genet.

[3] Wittkopp PJ, Carroll SB, Kopp A (2003). Evolution in black and white: genetic control of pigment patterns in Drosophila. Trends Genet.

[4] Shakhmantsir I, Massad NL, Kennell JA (2013). Regulation of cuticle pigmentation in drosophila by the nutrient sensing insulin and TOR signaling pathways. Dev Dyn.

[5] Granger NA, Ebersohl R, Sparks TC (2000). Pharmacological characterization of dopamine receptors in the corpus allatum of Manduca sexta larvae. Insect Biochem Mol Biol.

[6] Park D, Han M, Kim Y-C, Han K-A, Taghert PH (2004). Ap-let neurons—a peptidergic circuit potentially controlling ecdysial behavior in Drosophila. Dev Biol.

[7] Srivastava DP (2005). Rapid, nongenomic responses to ecdysteroids and catecholamines mediated by a novel Drosophila G-protein-coupled receptor. J Neurosci.

[8] Bai H, Zhu F, Shah K, Palli SR (2011). Large-scale RNAi screen of G protein-coupled receptors involved in larval growth, molting and metamorphosis in the red flour beetle. BMC Genomics.

[9] Meyer JM, Ejendal KFK, Avramova LV, Garland-Kuntz EE, Giraldo-Calderón GI, Brust TF (2012). A “Genome-to-Lead” Approach for Insecticide Discovery: Pharmacological Characterization and Screening of Aedes aegypti D1-like Dopamine Receptors. PLoS Negl Trop Dis.

[10] Neckameyera WS, Quinna WG (1989). Isolation and characterization of the gene for Drosophila tyrosine hydroxylase. Neuron.

[11] Livingstone MS, Tempel BL (1983). Genetic dissection of monoamine neurotransmitter synthesis in Drosophila. Nature.

[12] Eveleth DD, Gietzl RD, Spencer CA, Nargang FE, Hodgetts RB, Marsh JL (1986). Sequence and structure of the dopa decarboxylase gene of Drosophila: evidence for novel RNA splicing variants. EMBO J.

[13] Budnik V, White K (1987). Genetic dissection of dopamine and serotonin synthesis in the nervous system of Drosophila melanogaster. J Neurogenet.

[14] Valles AM, White K (1986). Development of serotonin-containing neurons in Drosophila mutants unable to synthesize serotonin. J Neurosci.

[15] Riemensperger T, Isabel G, Coulom H, Neuser K, Seugnet L, Kume K (2010). Behavioral consequences of dopamine deficiency in the Drosophila central nervous system. Proc Natl Acad Sci U S A.

[16] Yamamoto S, Seto ES (2014). Dopamine dynamics and signaling in Drosophila: an overview of genes, drugs and behavioral paradigms. Exp Anim.

[17] Gotzes F, Balfanz S, Baumann A (1994). Receptors & channels.: Primary structure and functional characterization of a Drosophila dopamine receptor with high homology to human D1/5 receptors. Recept Channels.

[18] Sugamori KS, Demchyshyn LL, McConkey F, Forte MA, Niznik HB (1995). A primordial dopamine D1-like adenylyl cyclase-linked receptor from Drosophila melanogaster displaying poor affinity for benzazepines. FEBS.

[19] Feng G, Hannan F, Reale V, Hon YY, Kousky CT, Evans PD (1996). Cloning and functional characterization of a novel dopamine receptor from Drosophila melanogaster. J Neurosci.

[20] Han K-A, Millar NS, Grotewiel MS, Davis RL (1996). DAMB, a Novel Dopamine Receptor Expressed Specifically in Drosophila Mushroom Bodies. Neuron.

[21] Ishimoto H, Takahashi K, Ueda R, Teiichi T (2005). G-protein gamma subunit 1 is required for sugar reception in Drosophila. EMBO J.

[22] Evans PD, Bayliss A, Reale V (2014). GPCR-mediated rapid, non-genomic actions of steroids: Comparisons between DmDopEcR and GPER1 (GPR30). Gen Comp Endocrinol.

[23] Inagaki HK, de-Leon SB-T, Wong AM, Jagadish S, Ishimoto H, Barnea G (2012). Visualizing Neuromodulation In Vivo: TANGO-Mapping of Dopamine Signaling Reveals Appetite Control of Sugar Sensing. Cell.

[24] Hearn MG, Ren Y, McBride EW, Reveillaud I, Beinborn M, Kopin AS (2002). A Drosophila dopamine 2-like receptor: molecular characterization and identification of multiple alternatively spliced variants. Proc Natl Acad Sci U S A.

[25] Draper I, Kurshan PT, McBride E, Jackson FR, Kopin AS (2007). Locomotor activity is regulated by D2-like receptors in Drosophila: an anatomic and functional analysis. Devel Neurobio.

[26] Kim YC, Lee HG, Han KA (2007). D1 dopamine receptor dDA1 is required in the mushroom body neurons for aversive and appetitive learning in Drosophila. J Neurosci.

[27] Andretica R, Kimc Y-C, Jonesa FS, Hanc K-A, Greenspan RJ (2008). Drosophila D1 dopamine receptor mediates caffeine-induced arousal. Proc Natl Acad Sci U S A.

[28] Seugnet L, Suzuki Y, Vine L, Gottschalk L, Shaw PJ (2008). D1 Receptor Activation in the Mushroom Bodies Rescues Sleep-Loss-Induced Learning Impairments in Drosophila. Curr Biol.

[29] Kong EC, Woo K, Li H, Lebestky T, Mayer N, Sniffen MR (2010). A pair of dopamine neurons target the D1-like dopamine receptor DopR in the central complex to promote ethanol-stimulated locomotion in Drosophila. PLoS One.

[30] Lebestky T, Chang J-SC, Dankert H, Zelnik L, Kim Y-C, Han K-A (2009). Two different forms of arousal in Drosophila Are oppositely regulated by the dopamine D1 receptor ortholog DopR via distinct neural circuits. Neuron.

[31] Bang S, Hyun S, Hong S-T, Kang J, Jeong K, Park J-J (2011). Dopamine signalling in mushroom bodies regulates temperature-preference behaviour in Drosophila. PLoS Genet.

[32] Mustard JA, Pham PM, Smith BH (2010). Modulation of motor behavior by dopamine and the D1-like dopamine receptor AmDOP2 in the honey bee. J Insect Physiol.

[33] Guo X, Ma Z, Kang L (2015). Two dopamine receptors play different roles in phase change of the migratory locust. Front Behav Neurosci.

[34] Friggi-Grelin F, Ich M, Birman S (2003). Tissue-specific developmental requirements of Drosophila tyrosine hydroxylase isoforms. Genesis.

[35] Monastirioti M (1999). Biogenic amine systems in the fruit fly Drosophila melanogaster. Microsc Res Tech.

[36] Neckameyer W, ODonnell J, Huang Z, Stark W (2001). Dopamine and Sensory Tissue Development in Drosophila melanogaster. Devel Neurobio.

[37] Birman S, Morgan B, Anzivino M, Hirsh A (1994). A novel and mojor isoform of tyrosine hydroxylase in Drosophila is generated by alternative RNA processing. J Biol Chem.

[38] Ueno T, Kume K (2014). Functional characterization of dopamine transporter in vivo using Drosophila melanogaster behavioral assays. Front Behav Neurosci.

[39] Waddell S (2013). Reinforcement signalling in Drosophila; dopamine does it all after all. Curr Opin Neurobiol.

[40] Abrieux A, Duportets L, Debernard S, Gadenne C, Anton S (2014). The GPCR membrane receptor, DopEcR, mediates the actions of both dopamine and ecdysone to control sex pheromone perception in an insect. Front Behav Neurosci.

[41] Mustard JA, Blenau W, Hamilton IS, Ward VK, Ebert PR, Mercer AR (2003). Analysis of two D1-like dopamine receptors from the honey bee apis mellifera reveals agonist-independent activity. Mol Brain Res.

[42] Mustard JA, Beggs KT, Mercer AR (2005). Molecular biology of the invertebrate dopamine receptors. Arch Insect Biochem Physiol.

[43] Larkin MA, Blackshields G, Brown NP, Chenna R, McGettigan PA, McWilliam H (2007). Clustal W and Clustal X version 2.0.. Bioinformatics.

[44] Duffy JB (2002). GAL4 system in Drosophila: A fly geneticist's swiss army knife. Genesis.

[45] Selcho M, Pauls D, Han K-A, Stocker RF, Thum AS (2009). The role of dopamine in Drosophila larval classical olfactory conditioning. PLoS One.

[46] Berry JA, Cervantes-Sandoval I, Nicholas EP, Davis RL (2012). Dopamine is required for learning and forgetting in Drosophila. Neuron.

[47] Cassar M, Issa A-R, Riemensperger T, Petitgas C, Rival T, Coulom HL (2015). A dopamine receptor contributes to paraquat-induced neurotoxicity in Drosophila. Hum Mol Genet.

[48] Yuan Q, Joiner WJ, Sehgal A (2006). A sleep-promoting role for the Drosophila serotonin receptor 1A. Curr Biol.

[49] Edgar R, Domrachev M, Lash AE (2002). Gene Expression Omnibus: NCBI gene expression and hybridization array data repository. Nucleic Acids Res.

[50] St Pierre SE, Ponting L, Stefancsik R, McQuilton P, the FlyBase Consortium (2014). FlyBase 102--advanced approaches to interrogating FlyBase. Nucleic Acids Res.

[51] Huang DW, Sherman BT, Lempicki RA (2009). Bioinformatics enrichment tools: paths toward the comprehensive functional analysis of large gene lists. Nucleic Acids Res.

[52] Buchon N, Broderick NA, Poidevin M, Pradervand S, Lemaitre B (2009). Drosophila intestinal response to bacterial infection: activation of host defense and stem cell proliferation. Cell Host and Microbe.

[53] Wang J, Duncan D, Shi Z, Zhang B (2013). WEB-based GEne SeT AnaLysis Toolkit (WebGestalt): update 2013. Nucleic Acids Res.

[54] Zhang B, Kirov S, Snoddy J (2005). WebGestalt: an integrated system for exploring gene sets in various biological contexts. Nucleic Acids Res.

[55] Erturk-Hasdemira D, Broemerb M, Leulierb FO, Lanec WS, Paquettea N, Hwanga D (2009). Two roles for the Drosophila IKK complex in the activation of Relish and the induction of antimicrobial peptide genes. Proc Natl Acad Sci U S A.

[56] Zhang K, Guo JZ, Peng Y, Xi W, Guo A (2007). Dopamine-mushroom body circuit regulates saliency-based decision-making in Drosophila. Science.

[57] Gorski SM, Chittaranjan S, Pleasance ED, Freeman JD, Anderson CL, Varhol RJ (2003). A SAGE approach to discovery of genes involved in autophagic cell death. Curr Biol.

[58] Scuderi A, Letsou A (2005). Amnioserosa is required for dorsal closure in Drosophila. Dev Dyn.

[59] Troppmann B, Balfanz S, Krach C, Baumann A, Blenau W (2014). Characterization of an invertebrate-type dopamine receptor of the american cockroach, periplaneta americana. IJMS.

[60] Gifford AN (1991). the dopamine and 5-hydroxytryptamine content of locust and cockroach salivary neurones. J Exp Biol.

[61] Baumann O, Dames P, Kuhnel D, Walz B (2002). Distribution of serotonergic and dopaminergic nerve fibers in the salivary gland complex of the cockroach Periplaneta americana. BMC Physiol.

[62] Šimo L, Koči J, Žitňan D, Park Y (2011). Evidence for D1 dopamine receptor activation by a paracrine signal of dopamine in tick salivary glands. PLoS One.

[63] Šimo L, Koči J, Kim D, Park Y (2014). Invertebrate specific D1-like dopamine receptor in control of salivary glands in the black-legged tick Ixodes scapularis. J Comp Neurol.

[64] Ali DW, Orchard I, Lange AB (1993). The aminergic control of locust (Locusta migratoria) salivary glands: evidence for dopaminergic and serotonergic innervation. J Insect Physiol.

[65] Hill CA, Fox AN, Pitts RJ, Kent LB, Tan PL, Chrystal MA (2002). G Protein-Coupled Receptors in Anopheles gambiae. Science.

[66] Nuss AB, Ejendal KFK, Doyle TB, Meyer JM, Lang EG, Watts VJ (2015). Dopamine receptor antagonists as New mode-of-action insecticide leads for control of aedes and Culex mosquito vectors. PLoS Negl Trop Dis.

[67] Davis MM, O'Keefe SL, Primrose DA, Hodgetts RB (2007). A neuropeptide hormone cascade controls the precise onset of post-eclosion cuticular tanning in Drosophila melanogaster. Development.

[68] Gelbart WM, Emmert DB (2013). FlyBase High Throughput Expression Pattern Data.

[69] Charles JP (2010). The regulation of expression of insect cuticle protein genes. Insect Biochem Mol Biol.

[70] Fechtel K, Fristrom DK, Fristrom JW (1989). Prepupal differentiation in Drosophila: distinct cell types elaborate a shared structure, the pupal cuticle, but accumulate transcripts in unique patterns. Development.

[71] Apple RT, Fristrom JW (1991). 20-hydroxyecdysone is required for, and negatively regulates, transcription of Drosophila pupal cuticle protein genes. Dev Biol.

[72] Doctor J, Fristrom DK, Fristrom JW (1985). The pupal cuticle of Drosophila: biphasic synthesis of pupal cuticle proteins in vivo and in vitro in response to 20-hydroxyecdysone. J Cell Biol.

[73] Wright LG, Chen T, Thummel CS, Guild GM (1996). Molecular characterization of the 71E late puff in Drosophila melanogaster reveals a family of novel genes. J Mol Biol.

[74] Crossgrove K, Bayer CA, Fristrom JW, Guild GM (1996). The Drosophila broad-complex early gene directly regulates late gene transcription during the ecdysone-induced puffing cascade. Dev Biol.

[75] Davis MB, Carney GE, Robertson AE, Bender M (2005). Phenotypic analysis of EcR-A mutants suggests that EcR isoforms have unique functions during Drosophila development. Dev Biol.

[76] Beggs KT, Tyndall JDA, Mercer AR (2011). Honey Bee dopamine and octopamine receptors linked to intracellular calcium signaling have a close phylogenetic and pharmacological relationship. PLoS One.

[77] Blenau W, Erber J, Baumann A (1998). Characterization of a dopamine D1 receptor from apis mellifera: cloning, functional expression, pharmacology, and mRNA localization in the brain. J Neurochem.

[78] Reale V, Hannan F, Hall LM, Evans PD (1997). Agonist-specific coupling of a cloned Drosophila melanogaster D1-like dopamine receptor to multiple second messenger pathways by synthetic agonists. J Neurosci.

[79] Conley JM, Meyer JM, Nuss AB, Doyle TB, Savinov SN, Hill CA (2015). Evaluation of AaDOP2 receptor antagonists reveals antidepressants and antipsychotics as novel lead molecules for control of the yellow fever mosquito, Aedes aegypti. J Pharmacol Exp Ther.

[80] Abdelsadik A, Roeder T (2010). Chronic activation of the epithelial immune system of the fruit fly's salivary glands has a negative effect on organismal growth and induces a peculiar set of target genes. BMC Genomics.

[81] Lee K-Z, Ferrandon D (2011). Negative regulation of immune responses on the fly. EMBO J.

[82] Åkerfelt M, Morimoto RI, Sistonen L (2010). Heat shock factors: integrators of cell stress, development and lifespan. Nat Rev Mol Cell Biol.

[83] Charroux B, Royet J (2012). Gut-microbiota interactions in non-mammals: what can we learn from Drosophila?. Semin Immunol.

[84] Storelli G, Defaye A, Erkosar B, Hols P, Royet J, Leulier F (2011). Lactobacillus plantarum promotes Drosophila systemic growth by modulating hormonal signals through TOR-dependent nutrient sensing. Cell Metab.

[85] Shin SC, Kim SH, You H, Kim B, Kim AC, Lee KA (2011). Drosophila microbiome modulates host developmental and metabolic homeostasis via insulin signaling. Science.

[86] Rynes J, Donohoe CD, Frommolt P, Brodesser S, Jindra M, Uhlirova M (2012). Activating transcription factor 3 regulates immune and metabolic homeostasis. Mol Cell Biol.

[87] Aparicio R, Neyen C, Lemaitre B, Busturia A (2013). dRYBP Contributes to the Negative Regulation of the Drosophila Imd Pathway. PLoS One.

[88] Ryu JH, Nam KB, Oh CT, Nam HJ, Kim SH, Yoon JH (2004). The homeobox gene caudal regulates constitutive local expression of antimicrobial peptide genes in Drosophila epithelia. Mol Cell Biol.

[89] Myllymaki H, Ramet M (2013). Transcription factor zfh1 downregulates Drosophila Imd pathway. Dev Comp Immunol.

[90] Lhocine N, Ribeiro PS, Buchon N, Wepf A, Wilson R, Tenev T (2008). PIMS modulates immune tolerance by negatively regulating Drosophila innate immune signaling. Cell Host and Microbe.

[91] Fernando MDA, Kounatidis I, Ligoxygakis P (2014). Loss of Trabid, a New Negative Regulator of the Drosophila Immune-Deficiency Pathway at the Level of TAK1, Reduces Life Span. PLoS Genet.

[92] Maillet F, Bischoff V, Vignal C, Hoffmann J, Royet J (2008). The Drosophila peptidoglycan recognition protein PGRP-LF blocks PGRP-LC and IMD/JNK pathway activation. Cell Host and Microbe.

[93] Tryselius Y, Samakovlis C, Kimbrell DA, Hultmark D (1992). CecC, a cecropin gene expressed during metamorphosis in Drosophila pupae. Eur J Biochem.

[94] Zhang Z, Palli SR (2009). Identification of a cis-regulatory element required for 20-hydroxyecdysone enhancement of antimicrobial peptide gene expression in Drosophila melanogaster. Insect Mol Biol.

[95] Broderick NA, Buchon N, Lemaitre B (2014). Microbiota-induced changes in Drosophila melanogaster host gene expression and Gut morphology. mBio.

[96] Levy F (2003). Proteomic Analysis of the Systemic Immune Response of Drosophila. Mol Cell Proteomics.

[97] Verleyen P, Baggerman G, D’Hertog W, Vierstraete E, Husson SJ, Schoofs L (2006). Identification of new immune induced molecules in the haemolymph of Drosophila melanogaster by 2D-nanoLC MS/MS. J Insect Physiol.

[98] Sirotkin K, Davidson N (1982). Developmentally regulated transcription from Drosophila melanogaster chromosomal site 67B. Dev Biol.

[99] Mason PJ, Hall LMC, Gausz J (1984). The expression of heat shock genes during normal development in Drosophila melanogaster (heat shock/abundant transcripts/developmental regulation). Mol Gen Genet.

[100] Irland R, Berger E, Sirotkin K, Yund MA, Osterbur D, Fristrom JW (1982). Ecdysterone induces the transcription of four heat-shock genes in Drosophila S3 cells and lmaginal discs. Dev Biol.

[101] Takahashi KH, Rako L, Takano-Shimizu T, Hoffmann AA, Lee SF (2010). Effects of small Hsp genes on developmental stability and microenvironmental canalization. BMC Evol Biol.

[102] Ayme A, Tissieres A (1985). Locus 67B of Drosophila melanogaster contains seven, not four, closely related heat shock genes. EMBO J.

[103] Krebs RA, Feder ME (1997). Deleterious consequences of Hsp70 overexpression in Drosophila melanogaster larvae. Cell Stress Chaperones.

[104] Badenhorst P (2005). The Drosophila nucleosome remodeling factor NURF is required for Ecdysteroid signaling and metamorphosis. Genes Dev.

[105] Zraly CB, Middleton FA, Dingwall AK (2006). Hormone-response genes Are direct in vivo regulatory targets of Brahma (SWI/SNF) complex function. J Biol Chem.

[106] Tadevosyan A, Vaniotis G, Allen BG, Herbert TE, Nattel S (2012). G protein-coupled receptor signalling in the cardiac nuclear membrane: evidence and possible roles in physiological and pathophysiological function. J Physiol.

[107] Vaniotis G, Del Duca D, Trieu P, Rohlicek CV, Herbert TE, Allen BG (2011). Cellular signalling. Cell Signal.

[108] Boivin B, Vaniotis G, Allen BG, Hébert TE (2008). G protein-coupled receptors in and on the cell nucleus: a New signaling paradigm?. J Receptors and Signal Transduction.

[109] Wright CD, Wu SC, Dahl EF, Sazama AJ, O'Connell TD (2012). Nuclear localization drives α1-adrenergic receptor oligomerization and signaling in cardiac myocytes. Cell Signal.

[110] Joyal J-S, Bhosle VK, Chemtob S (2015). Subcellular G-protein coupled receptor signaling hints at greater therapeutic selectivity. Expert Opin Ther Targets.

[111] Patel N, Itakura T, Gonzalez JM, Schwartz SG, Fini ME (2013). GPR158, an orphan member of G protein-coupled receptor family C: glucocorticoid-stimulated expression and novel nuclear role. PLoS One.

[112] Newby L, Jackson RF (1991). Drosophila ebony mutants have altered circadian activity rhythms but normal eclosion rhythms. J Neurogenet.

[113] Brand AH, Perrimon N (1993). Targeted gene expression as a means of altering cell fates and generating dominant phenotypes. Development.

[114] Rubin GM, Spradling AC (1982). Genetic transformation of Drosophila with transposable element vectors. Science.

[115] Benjamini Y, Drai D, Elmer G, Kafkafi N, Golani I (2001). Controlling the false discovery rate in behavior genetics research. Behav Brain Res.

[116] Wang P-H, Wan D-H, Gu Z-H, Qiu W, Chen Y-G, Weng S-P (2013). Analysis of expression, cellular localization, and function of three Inhibitors of Apoptosis (IAPs) from litopenaeus vannamei during WSSV infection and in regulation of antimicrobial peptide genes (AMPs). PLoS One.

[117] Szklarczyk D, Franceschini A, Kuhn M, Simonovic M, Roth A, Minguez P (2010). The STRING database in 2011: functional interaction networks of proteins, globally integrated and scored. Nucleic Acids Res.

[118] Watts RJ, Schuldiner O, Perrino J, Larsen C, Luo L (2004). Glia engulf degenerating axons during developmental axon pruning. Curr Biol.

[119] Harwood BN, Fortin JP, Gao K, Chen C, Beinborn M, Kopin AS (2013). Membrane tethered bursicon constructs as heterodimeric modulators of the Drosophila G protein-coupled receptor rickets. Mol Pharmacol.

[120] Dietzl G, Chen D, Schnorrer F, Su K-C, Barinova Y, Fellner M (2007). A genome-wide transgenic RNAi library for conditional gene inactivation in Drosophila. Nature.

[121] Wodarz A, Hinz U, Engelbert M, Knust E (1995). Expression of crumbs confers apical character on plasma membrane domains of ectodermal epithelia of Drosophila. Cell.

[122] Choi C, Cao G, Tanenhaus AK, McCarthy EV, Jung M, Schleyer W (2012). Autoreceptor control of peptide/neurotransmitter corelease from PDF neurons determines allocation of circadian activity in Drosophila. Cell Rep.

