# Supplementary material for: A critical role for the Drosophila dopamine D1-like receptor Dop1R2 at the onset of metamorphosis
Source: BMC Dev Biol. 2016 May 16;16:15. doi: 10.1186/s12861-016-0115-z (PMC4868058; doi:10.1186/s12861-016-0115-z)
Supplement: Additional file 1: Figure S1. — dsDop1R2 RNAi constructs. Sequences of the three RNAi constructs utilized in the present study, and alignment on Dop1R2-RB mRNA GenBank reference sequence. The RNAi sequences include those used to generate Vienna Drosophila RNAi Center (VDRC) stocks 3391-GD (FBst0460369)/construct 703 and 105324-KK (FBst0477151)/construct 110947, as well as Tufts Medical Center (TMC) Dop1R2 lines (Materials and Methods). (PDF 120 kb) [file 12861_2016_115_MOESM1_ESM.pdf]

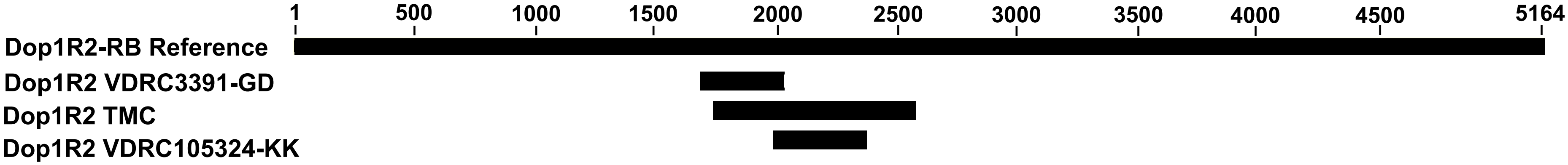

Dop1R2 VDRC3391-GD

CTGCGAGGGATGGCGAGATGCCCGCCTACAAGTGCACCTTCACTGAGCACCTAGGCTATCTGGTCTTCTCGTCGACGATA
TCCTTCTACCTGCCGCTTCTAGTGATGGTCTTCACCTACTGTCGCATCTACAGGGCAGCCGTCATCCAGACGAGATCTCT
TAAGATTGGAACCAAGCAGGTGCTCATGGCCTCCGGGGAACTGCAGCTCACATTGCGCATTCATCGTGGTGGCACTACGC
GGGATCAGCAAAACCAGGTCTCCGGAGGAGGAGGTGGCGGAGGAGGAGGTGGCGGTGGCGGAGGATCTCTGAGCCACTCG
CACTCCCATTCGCAC

Dop1R2 TMC

TCTGGTCTTCTCGTCGACGATATCCTTCTACCTGCCGCTTCTAGTGATGGTCTTCACCTACTGTCGCATCTACAGGGCAG
CCGTCATCCAGACGAGATCTCTTAAGATTGGAACCAAGCAGGTGCTCATGGCCTCCGGGGAACTGCAGCTCACATTGCGC
ATTCATCGTGGTGGCACTACGCGGGATCAGCAAAACCAGGTCTCCGGAGGAGGAGGTGGCGGAGGAGGAGGTGGCGGTGG
CGGAGGATCTCTGAGCCACTCGCACTCCCATTCGCACCACCATCATCACAATCACGGCGGTGGCACGACGACCTCCACGC
CGGAGGAGCCGGATGATGAGCCGCTATCCGCTCTGCATAACAACGGACTGGCACGCCATCGGCACATGGGCAAGAACTTC
TCGCTGTCCAGGAAACTGGCGAAGTTCGCCAAGGAGAAGAAAGCGGCCAAGACGCTGGGCATCGTGATGGGCGTGTTCAT
CATCTGTTGGCTGCCCTTCTTCGTGGTCAACCTGCTGTCTGGGTTCTGCATCGAGTGCATCGAGCACGAGGAGATCGTCT
CGGCAATCGTCACCTGGCTCGGCTGGATCAACTCCTGCATGAATCCTGTGATTTACGCCTGCTGGAGCAGGGACTTTCGC
AGGGCCTTTGTGCGTCTGCTGTGCATGTGCTGTCCACGCAAGATTCGCCGCAAGTACCAGCCCACGATGCGTTCCAAATC
GCAGAGATTCGCGACGCGGCGCTGCTACTCGACCTGCTCGCTGCACGGCATTCAGCACGTGCGACACAACTCCTGCGAGC
AGACCTACATATAGTTTAGCGTAGA

Dop1R2 VDRC105324-KK

GCAGGCGTAAATCACAGGATTCATGCAGGAGTTGATCCAGCCGAGCCAGGTGACGATTGCCGAGACGATCTCCTCGTGCT
CGATGCACTCGATGCAGAACCCAGACAGCAGGTTGACCACGAAGAAGGGCAGCCAACAGATGATGAACACGCCCATCACG
ATGCCCAGCGTCTTGGCCGCTTTCTTCTCCTTGGCGAACTTCGCCAGTTTCCTGGACAGCGAGAAGTTCTTGCCCATGTG
CCGATGGCGTGCCAGTCCGTTGTTATGCAGAGCGGATAGCGGCTCATCATCCGGCTCCTCCGGCGTGGAGGTCGTCGTGC
CACCGCCGTGATTGTGATGATGGTGGTGCGAATGGGAGTGCGAGTGGCTCAGAGAT
