# Supplementary figures and images for: A critical role for the Drosophila dopamine D1-like receptor Dop1R2 at the onset of metamorphosis
Source: BMC Dev Biol. 2016 May 16;16:15. doi: 10.1186/s12861-016-0115-z (PMC4868058; doi:10.1186/s12861-016-0115-z)

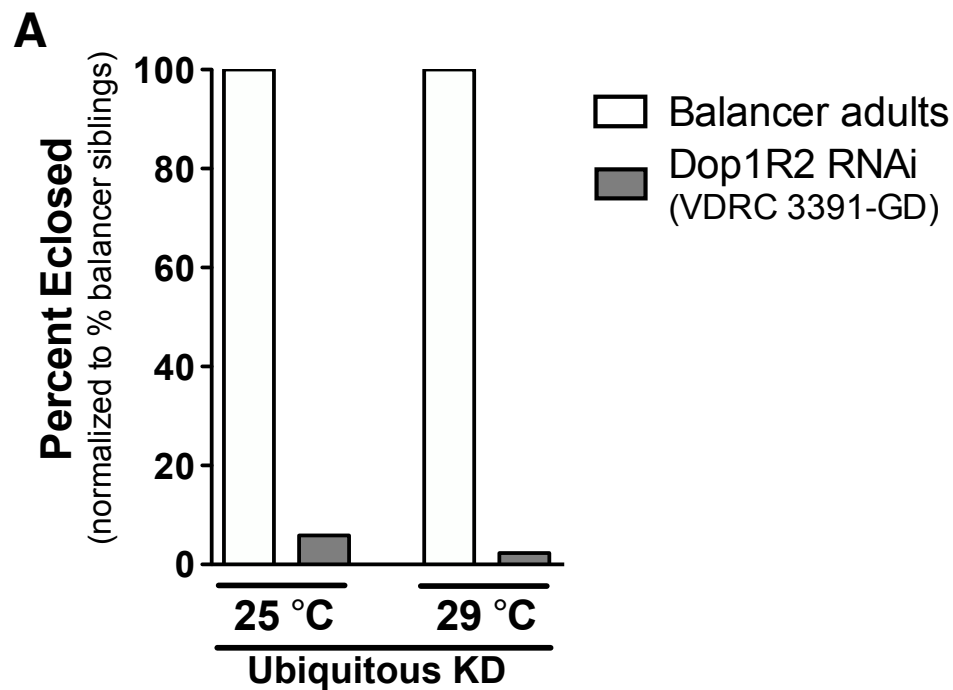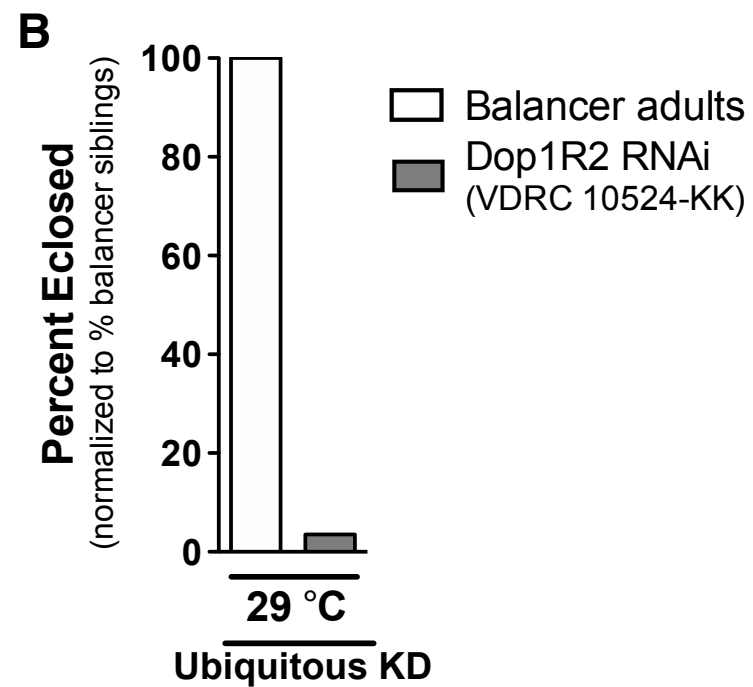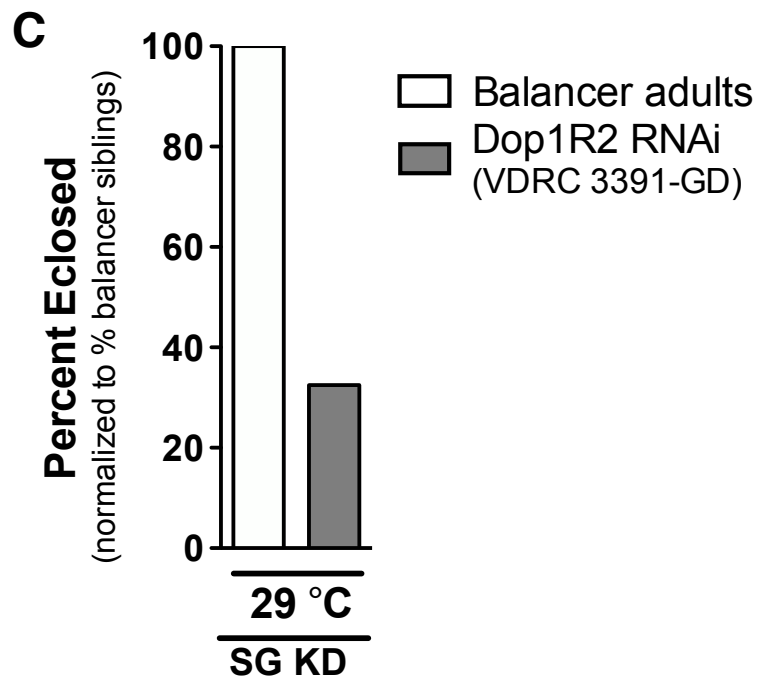

Supplement: Additional file 2: Figure S2. — dsDop1R2 knockdown-induced lethality is recapitulated with alternate RNAi constructs. (A) and (B) Ubiquitous knockdown of Dop1R2. (A) VDRC 3391-GD (genotype: w1118;UAS-dsDop1R2/+;Act5C-Gal4/+) results in 98 % lethality at 29 °C (n = 58) and 94 % lethality at 25 °C, or viability (n = 44). (B) VDRC 105324-KK (genotype: w1118;TM6B/+;UAS-dsDop1R2/+) results in 97 % lethality at 29 °C (n = 59), compared to control balancer siblings (genotypes: w1118;CyO/+;UAS-dsDop1R2/+ and w1118;CyO/UAS-dsDop1R2, respectively). All male escaper flies (n = 4) obtained when using the VDRC 3391-GD RNAi construct exhibited the hypomelanization phenotype (described in Fig. 7). (C) Salivary gland/amnioserosa targeted knockdown of Dop1R2 VDRC 3391-GD results in 68 % lethality at 29 °C in experimental flies (genotype: w1118;UAS-dsDop1R2/+;P{GawB}c729-GAL4/+), compared to controls (genotype: w1118;P{GawB}c729-Gal4/+) (n = 53). VDRC Dop1R2 knockdown stocks: 3391-GD (FBst0460369) and 105324-KK (FBst0477151). Driver stocks: Act5C-GAL4 (FBst0003954), P{GawB}17A-GAL4 (FBst0008474) and P{GawB}c729-GAL4 (FBst0006983). (PDF 230 kb) [file 12861_2016_115_MOESM2_ESM.pdf]

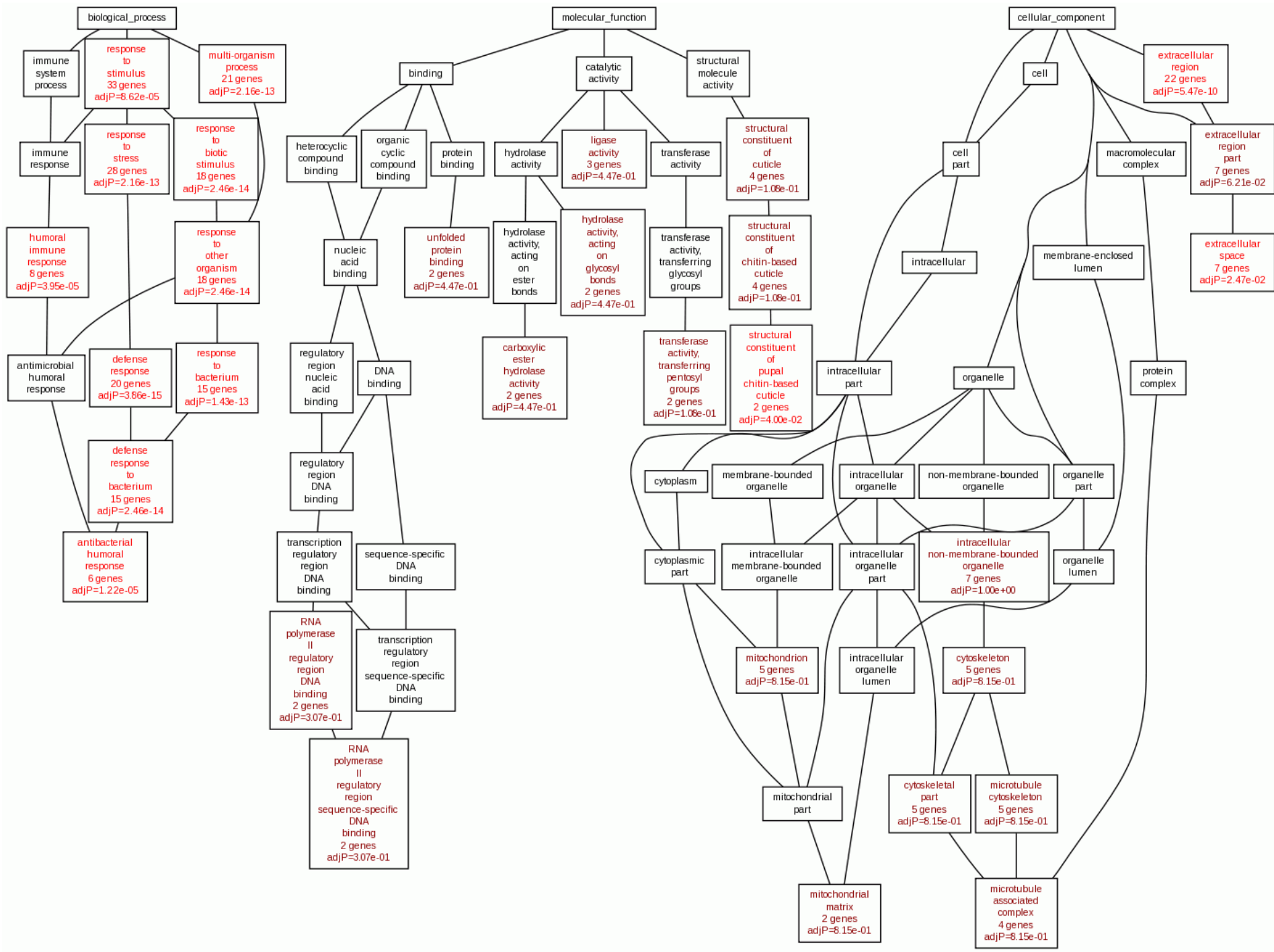

Supplement: Additional file 5: Figure S3. — WEB-based GEne SeT AnaLysis (WEBGestalt). Analysis of dsDop1R2 differentially expressed genes reveals enrichment in GO categories categorized by biological process, molecular function and cellular component. The top 10 GO categories that have a Benjamini corrected p-value of < 0.05 (red) and p-value > 0.05 (brown), as well as the non-enriched parents (black), are depicted. Each node provides: GO category, gene number in category and the adjusted p-value indicating the significance of enrichment. (PDF 207 kb) [file 12861_2016_115_MOESM5_ESM.pdf]

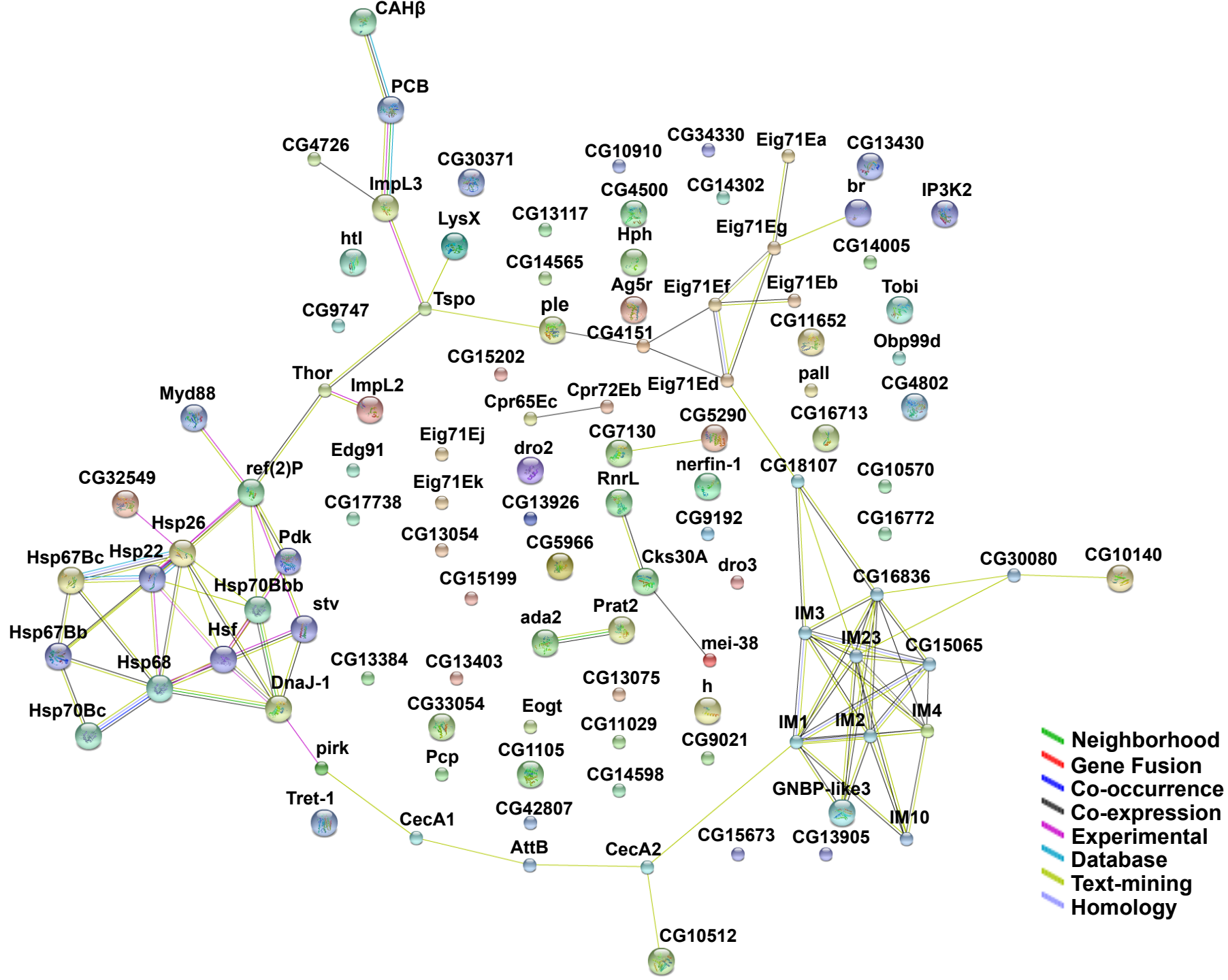

Supplement: Additional file 6: Figure S4. — STRING analysis reveals protein-protein interactions. Interactions indicated by connecting lines. Interactions predicted based on genomic content high throughput expression, co-expression and/or text-mining via STRING database (version 10) [117]. Legend indicates resource used in interaction prediction. (PDF 410 kb) [file 12861_2016_115_MOESM6_ESM.pdf]

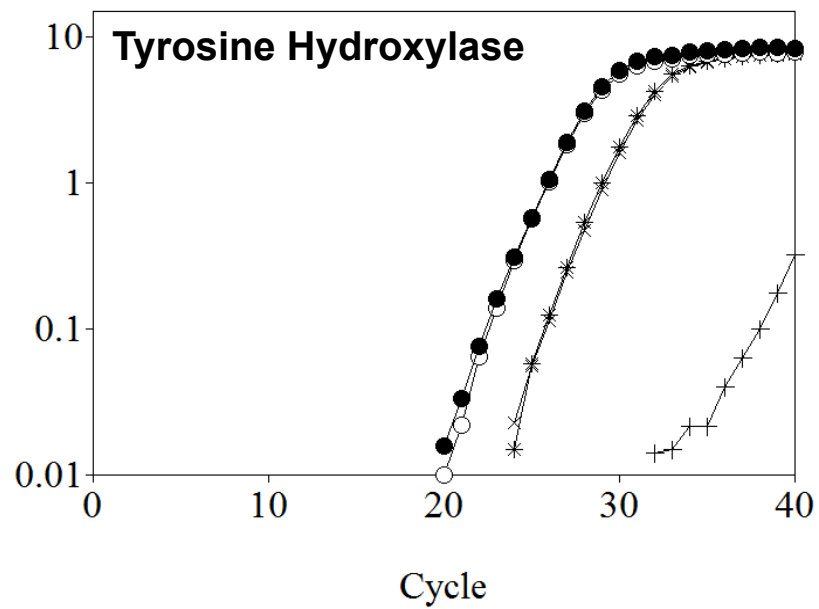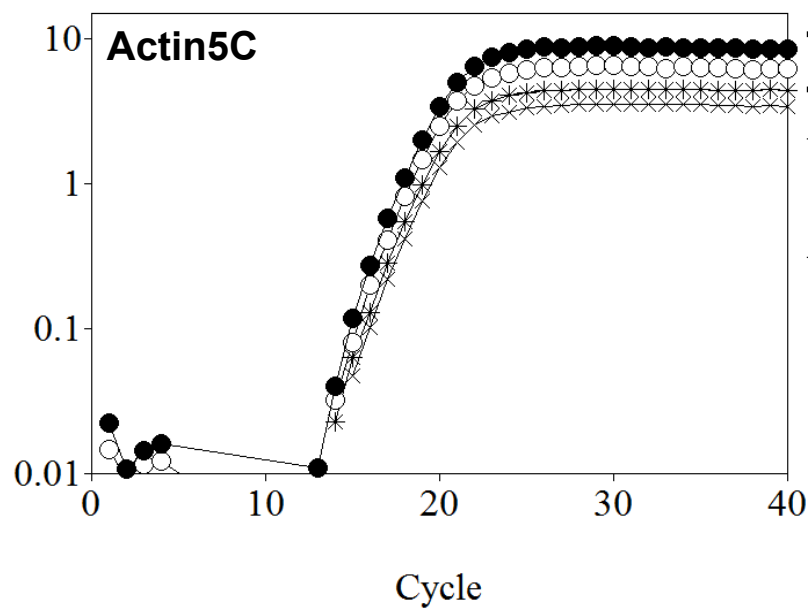

Supplement: Additional file 7: Figure S5. — Tyrosine hydroxylase expression is increased in dsDop1R2 pupae. Dop1R2 knockdown pupae with the genotype w1118;UAS-dsDop1R2/+;Act5C-GAL4/+) exhibit increased TH transcript levels compared to controls (genotype: w1118;UAS-dsDop1R2/+;TM6B/+). Four-fold difference (i.e., two cycles of amplification) in TH expression is observed in Dop1R2 RNAi vs. controls using two independent biological replicates. Driver stock: Act5C-GAL4 (FBst0003954). (PDF 82 kb) [file 12861_2016_115_MOESM7_ESM.pdf]

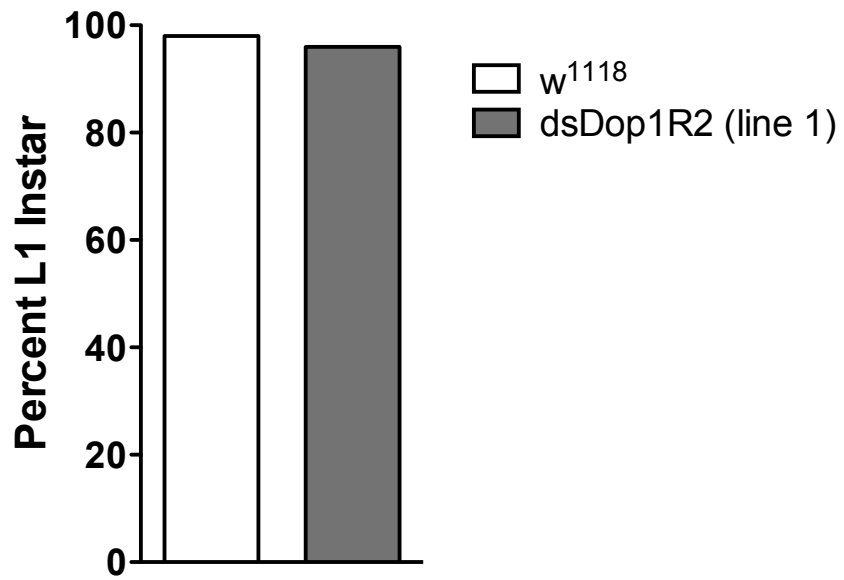

Supplement: Additional file 8 : Figure S6. — Progression from egg to L1 instar. Dop1R2 RNAi (line 1) or w1118 flies were crossed with the P{GawB}332.3 driver line (GAL4 expressed in the salivary glands and amnioserosa [121]) to assess completion of embryogenesis. dsDop1R2 flies (genotype: w1118;UAS-dsDop1R2/P{GawB}332.3-GAL4) showed similar progression into L1 compared to controls (genotype: w1118;P{GawB}332.3-GAL4/+) (n = 50). Driver stock: P{GawB}332.3-GAL4 (FBst0005398). (PDF 26 kb) [file 12861_2016_115_MOESM8_ESM.pdf]

## Brightfield 100X

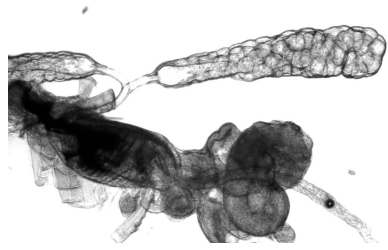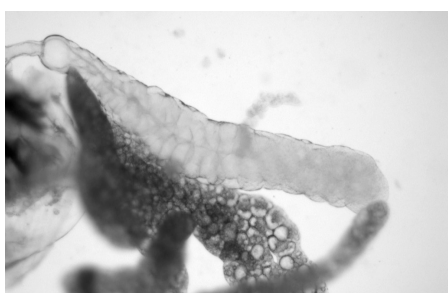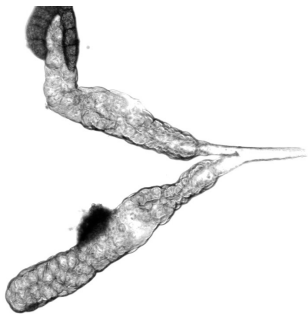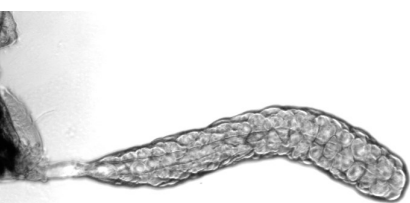

## GFP 100X

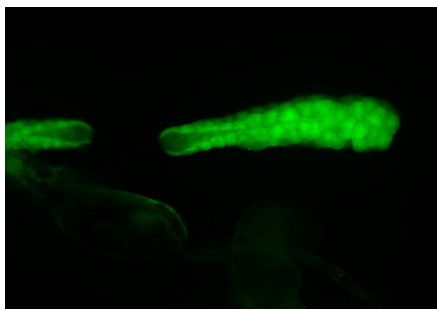

w<sup>\*</sup>;P{GawB}17A

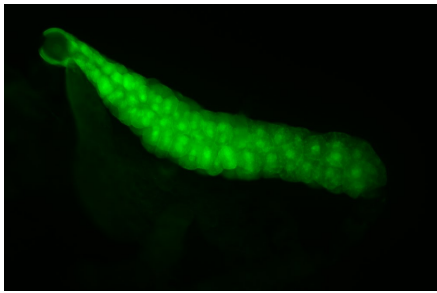

w<sup>\*</sup>;P{GawB}c729

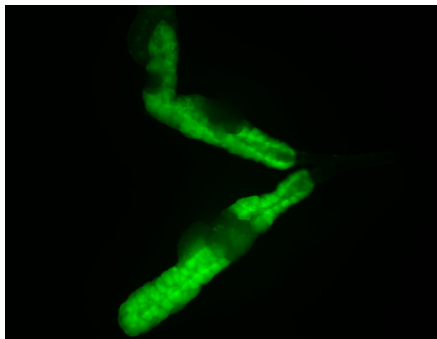

w<sup>\*</sup>;P{GawB}332.3

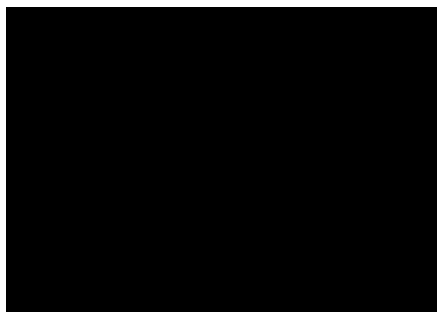

w<sup>1118</sup>

Supplement: Additional file 10: Figure S8. — Confirmation of salivary gland expression induced by the GAL4 drivers that were utilized. To confirm GAL4 expression in salivary glands, the UAS-GFP responder stock: w*;P{UAS-2xEGFP}AH2 (FBst0006874) was crossed with either (i) FBst0005398, (ii) FBst0008474 or (iii) FBst0006983, driver lines (see Table 1). The genotype of the corresponding progeny is (i) w1118;UAS-EGFP/P{GawB}332.3-GAL4, (ii) w1118;UAS-EGFP/P{GawB}17A-GAL4 and (iii) w1118;UAS-EGFP/P{GawB}c729-GAL4. The UAS-GFP responder stock was also crossed with w1118 to generate w1118;UAS-EGFP/+ controls. All three drivers tested resulted in marked GFP expression in the salivary glands. No overlapping fluorescence was detected in other tissue/cell type. Control flies showed dull (background) fluorescence only. All images, magnification: 100X, image exposure: 5 msec. (PDF 2465 kb) [file 12861_2016_115_MOESM10_ESM.pdf]

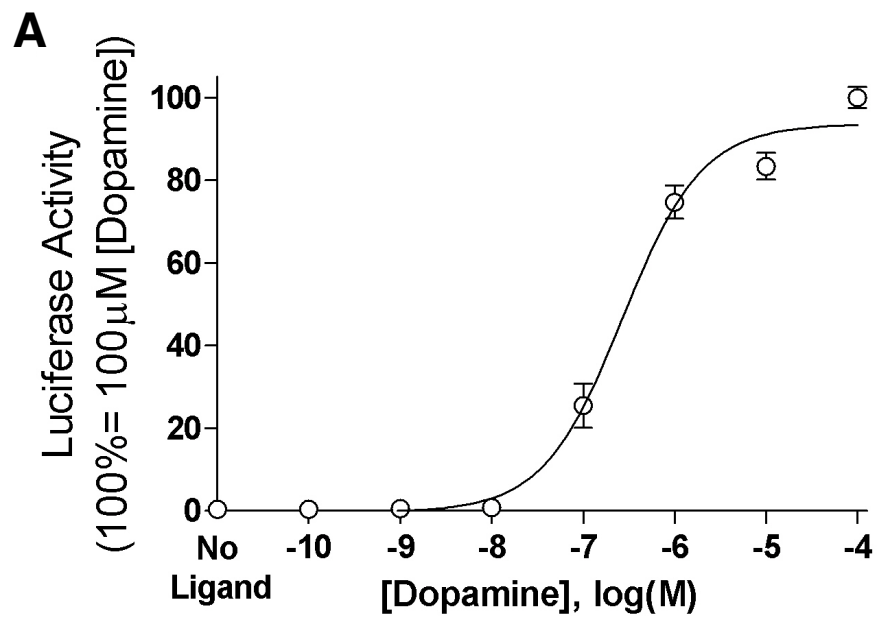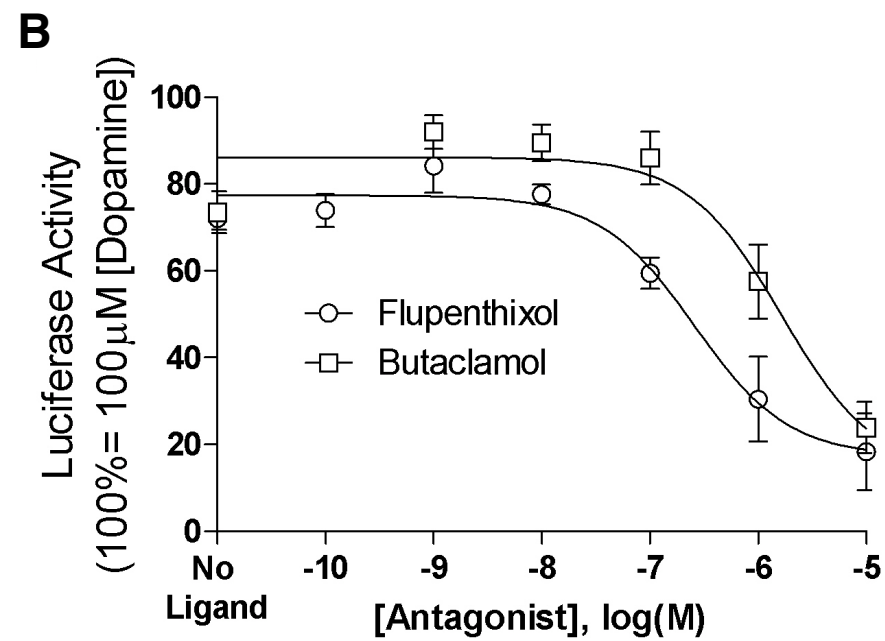

Supplement: Additional file 11: Figure S9. — Dop1R2 is stimulated by dopamine and antagonized by two known small molecules in vitro. Human Embryonic Kidney cells (HEK293 cells) were transiently co-transfected in a 96-well plate assay with plasmids encoding Drosophila Dop1R2 receptor and a luciferase reporter gene. (A) Increasing concentrations of dopamine activates the receptor (EC50 = 2.7 × 10^−7^ M). (B) Stimulation of the Dop1R2 receptor by dopamine (100 μM) is inhibited with increasing concentrations of either flupenthixol dihydrochloride (IC50 = 2.6 × 10^−7^ M) or butaclamol (IC50 = 21.6 × 10−7 M). Data represent the mean ± the Standard Error of the Mean (SEM) from three independent experiments, each performed in triplicate. Methods for this assay are as previously described [119]. (PDF 227 kb) [file 12861_2016_115_MOESM11_ESM.pdf]

**A**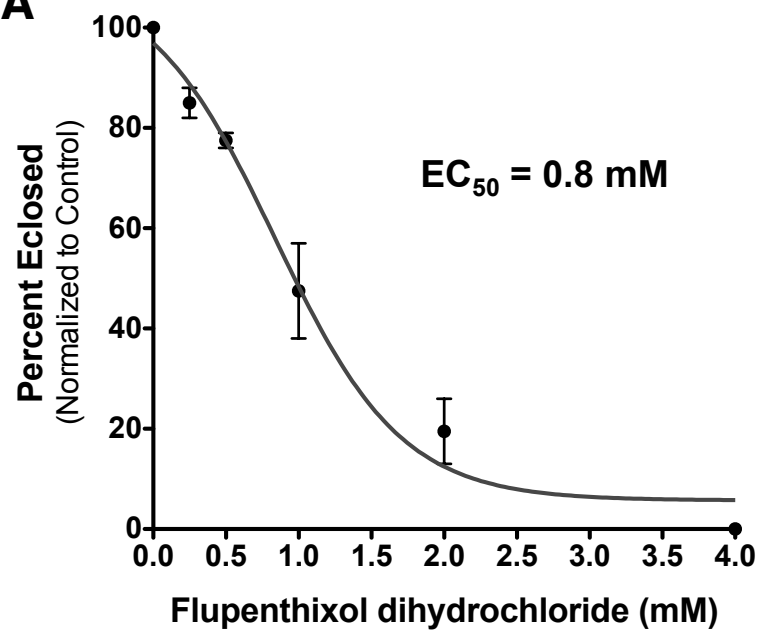**B**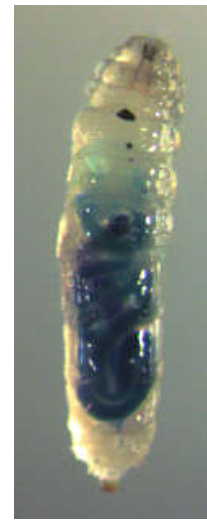**C**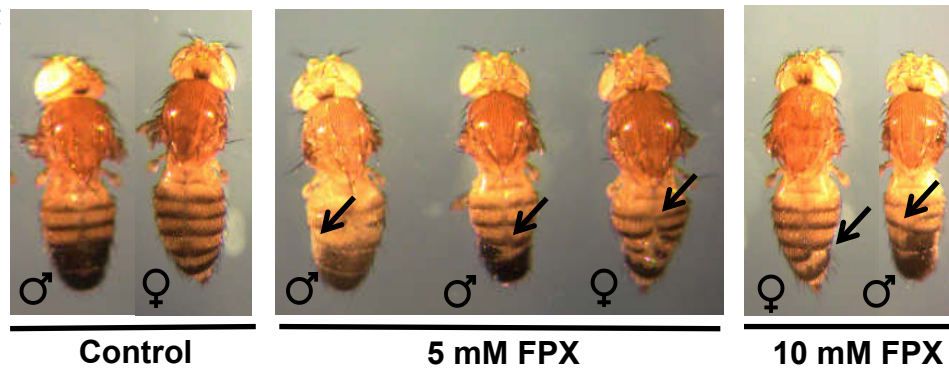

Supplement: Additional file 12: Figure S10. — Exposure of Drosophila melanogaster (w1118) larvae to flupenthixol dihydrochloride results in increased lethality and developmental defect. (A) Assessment of adult eclosion following larval exposure to flupenthixol dihydrochloride reveals a concentration-dependent effect (EC50 = 0.8 mM). (B-C) Feeding flupenthixol (5 mM or 10 mM, 0.1 % fast green dye, in H2O) to L3 larvae (B) results in cuticle and melanization defects, in 13 % 10 % of adults, respectively. These defects are not observed in control flies (the corresponding larvae were fed 0.1 % fast green dye in H2O, only). (C) Images are showing two day old adults (5 days post-exposure onset). n = 30 larvae per concentration, three independent replicates. (PDF 459 kb) [file 12861_2016_115_MOESM12_ESM.pdf]
