# Supplementary material for: A critical role for the Drosophila dopamine D1-like receptor Dop1R2 at the onset of metamorphosis
Source: BMC Dev Biol. 2016 May 16;16:15. doi: 10.1186/s12861-016-0115-z (PMC4868058; doi:10.1186/s12861-016-0115-z)

A Appearance:

Predicted BAF1/ABF1 chromatin reorganizing factor motif	
Position	337..380
Alignment<br>Query Database	SGGGGGGGGGGGGGGSLSHSHSHSHHHHHNHGGGTTTSTPEEPD<br>NEDGSPGGGSGDEGGHHHGHNMHAHHHHHHHHNGHTNGHGLAEDO
Score	46.3
E-value	3e-05

Sequence:

MVDDNGSSPEVEGAEGAGAPLLALLRVDGLNQTQTRSPSPSFFGSYNISEDVYFYFNGLPTSTELVLNATTSATSATLSP
AMVATGGGGTTTPEPDLSEFLEALPNDRVGLLAFLFLFSFATVFGNSLVILAVIRERYLHTATNYFITSLAVADCLVGLVV
MPFSALYEVLENTWFFGTDWCDIWRSLDVLFSTASILNLCVISLDRYWAITDPFSYPMRMTVKRAAGLIAAVWICSSAIS
FPAIVWWRAARDGEMPAYKCTFTEHLGYLVFSSTISFYLPLLVMVFTYCRIYRAAVIQTRSLKIGTKQVLMASGELQLTLR
IHRGGTTRDQQNQVSGGGGGGGGGGGGGGSLSHSHSHSHHHHHNHGGGTTTSTPEEPDDEPLSALHNNGLARHR
HMGKNFSLSRKLAKFAKEKKAAKTLGIVMGVFIICWLPFFVVNLLSGFCIECIEHEEIVSAIVTWLGWINSCMNPVIYAC
WSRDFRRAFVRLLCMCCPRKIRRKYQPTMRSKSQRFATRRCYSTCSLHGIQHVRHNSCEQTYI

Description: pfam04684, BAF1_ABF1, BAF1 / ABF1 chromatin reorganizing factor. ABF1 is a sequence-specific DNA binding protein involved in transcription activation, gene silencing and initiation of DNA replication. ABF1 is known to remodel chromatin, and it is proposed that it mediates its effects on transcription and gene expression by modifying local chromatin architecture. These functions require a conserved stretch of 20 amino acids in the C-terminal region of ABF1 (amino acids 639 to 662 *S. cerevisiae*). The N-terminal two thirds of the protein are necessary for DNA binding, and the N-terminus (amino acids 9 to 91 in *S. cerevisiae*) is thought to contain a novel zinc-finger motif which may stabilize the protein structure.

B

Predicted bipartite NLS in query sequence		
Pos.	Sequence	Score
391	GLARHRHMGKNFSLSRKLAKFAKEKKAAKT	6.4

MVDDNGSSPEVEGAEGAGAPLLALLRVDGLNQTQTRSPSPSFFGSYNISEDVYFYFNGLPTSTELVLNATTSATSATLSPAMVATGGGGTTTPEPDLSEFLEALPNDRVGLLAFLFLFSFATVFGNSLVIL
AVIRERYLHTATNYFITSLAVADCLVGLVVMPFSALYEVLENTWFFGTDWCDIWRSLDVLFSTASILNLCVISLDRYWAITDPFSYPMRMTVKRAAGLIAAVWICSSAISFPAIVWWRAARDGEMPAYK
CTFTEHLGYLVFSSTISFYLPLLVMVFTYCRIYRAAVIQTRSLKIGTKQVLMASGELQLTLRIHRGGTTRDQQNQVSGGGGGGGGGGGGGGSLSHSHSHSHHHHHNHGGGTTTSTPEEPDDEPLSALH
NNGLARHRHMGKNFSLSRKLAKFAKEKKAAKTLGIVMGVFIICWLPFFVVNLLSGFCIECIEHEEIVSAIVTWLGWINSCMNPVIYACWSRDFRRAFVRLLCMCCPRKIRRKYQPTMRSKSQRFATRRC
YSTCSLHGIQHVRHNSCEQTYI

C **Selected Dop1R2 protein region**

Predicted BAF1/ABF1 chromatin reorganizing factor motif ——

Predicted bipartite NLS ----

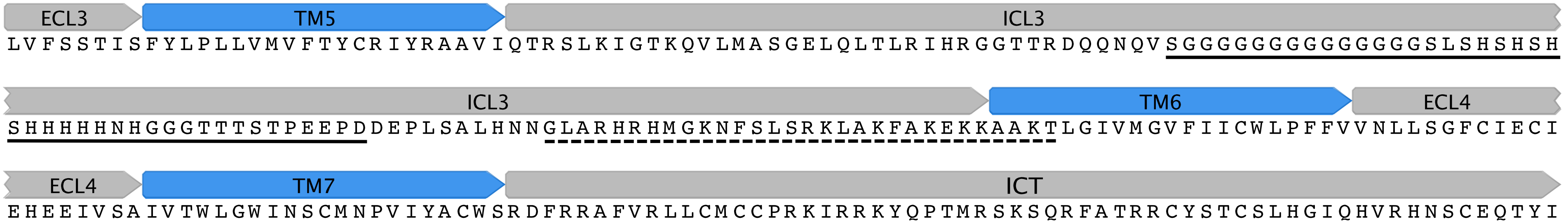

Supplement: Additional file 13: Figure S11. — Dop1R2 sequence motif. (A) GenomeNet motif analysis via (http://www.genome.jp/tools/motif/) reveals homology to BAF1/ABF1 chromatin reorganizing factor. (B) Sequence analysis via cNLS mapper (http://nls-mapper.iab.keio.ac.jp/cgi-bin/NLS_Mapper_form.cgi) reveals the presence of a bipartite nuclear localization signal. (C) The predicted BAF1/ABF1 chromatin reorganization factor motif (straight line) and a predicted bipartite nuclear localization signal (dotted line) are shown on the corresponding Dop1R2 protein sequence. TM: transmembrane, ECL: extracellular loop, ICL: intracellular loop, ICT: intracellular tail. (PDF 245 kb) [file 12861_2016_115_MOESM13_ESM.pdf]
