# Supplementary material for: A critical role for the Drosophila dopamine D1-like receptor Dop1R2 at the onset of metamorphosis
Source: BMC Dev Biol. 2016 May 16;16:15. doi: 10.1186/s12861-016-0115-z (PMC4868058; doi:10.1186/s12861-016-0115-z)
Supplement: Additional file 14: Table S1. — Primer sequences. (DOC 44 kb) [file 12861_2016_115_MOESM14_ESM.doc]

Table S1. Primer sequences.

| **Gene** | **Forward Sequence (5’ -> 3’)** | **Reverse Sequence (5’ -> 3’)** |
| --- | --- | --- |
| Dop1R2-RA/C | CAAATCGCAGTGTCACGTGG | GTGCTGCAACTGCTCAGCT |
| Dop1R2-RB | CCTGCGAGCAGACCTACATA | CGTGACACTTGCCATTGACT |
| Dop1R2 (in/in) | CACCTGGCTCGGCTGGATCAACT | CGACTTGGAACGCATCGTG |
| Dop1R2 (in/out) | CACGGATCCCTTTAGCTATC | CACCTGCTTGGTTCCAATCT |
| Act5C (set 1) | CAGCCAGCAGTCGTCTAATCC | CGACAACCAGAGCAGCAACTT |
| Act5C (set 2) | AACGGCTCTGGCATGTGC | ACTGGGTCATCTTCTCACGGT |
| repo | GTTCCTCCACGGTGGTTAAT | AGTAAAGGTTCTCGTCTTC |
| sgs5 | GATTGCCACAGAGAGATTCTACAG | CTGAATCCACCTCACTTAGAA |
| TH (set 1) | AGTTGCAGGAGATGTCCGAC | CTTGCAGAGACCGAACTCAA |
| TH (set 2) | AGTTGCAGGAGATGTCCGAC | AAGCTCTCGGCCACATAGTA |
| Dop2R (set 1) | CGAGCTGAGAGTGGTGGAC | GCTTGGCGTACTTTATTGGC |
| Dop2R (set 2) | GCCATGAAGCCATTGTCCTT | GTCGACGTTGTAGTACCTG |
| Oct-TyrR | TTGCATACAGGTCTGCGTGA | ATGTAGCCCAGCCAGGTGA |
| Oamb | AACATCAAGGCGCAGGTGAA | TTCAAGGAGACGGACTGGC |
| 5-HT1A | CAGAGCCACGTAGCCGACA | CGGAATCGCTGATCTGGCA |
| Dop1R1 | GCACCGGATTCTCCACGAAT | CCGGTTCCTCACCAACTATT |
| CecA2 | ACCACCATGAACTTCTACAACA | GGTTAACCTCGAGCAGTGG |
| Hsp67Bc | GGTCGTCGGTTCAACGAAC | GACGGTCAGTTCACCTGGC |
| Hsp70Bc | GTGAACACGTCGCTAAGCG | CCCTGGTCATTGGCGATAATCT |
| Edg91 | GAGTTGTATGCTGGCCCTT | GAGTAACCTCCTCCTGGATAGT |
| LysX | GGTGTTTCTCGTGACCAGTT | CTGTTGACCCAGGACCTTTAG |
| Dro2 | TTTCGTCTTCCTGGCTGTG | AGTATGGATTCAGCATCCTTCG |
| Dro3 | CTATCTGGAACTTTCGGAGGTC | CCTGAAAGGCAATGCTTACG |
| Cpr72Eb | GTTCACCACACTAGCAGCG | GGGCAGATTGGAGGTTACATGG |
| Rel | GAAGTTCCGCTTTCGGTACAA | GCCGCACCTGGTTCAAG |
