# Supplementary material for: A critical role for the Drosophila dopamine D1-like receptor Dop1R2 at the onset of metamorphosis
Source: BMC Dev Biol. 2016 May 16;16:15. doi: 10.1186/s12861-016-0115-z (PMC4868058; doi:10.1186/s12861-016-0115-z)
Supplement: Additional file 9: Figure S7. — dsDop1R2 knockdown-induced lethality is recapitulated with alternate salivary gland drivers. The wing and melanization phenotype is also observed in flies that survive to adulthood (A) Knockdown of Dop1R2 using a second larval salivary gland driver line (i.e., P{GawB}17A-GAL4; FBst0008474, which also targets glia and the proventriculus) results in semi-lethality in corresponding progeny. At 29 °C, 48.1 % of dsDop1R2 flies (genotype: w1118;UAS-dsDop1R2/P{GawB}17A-GAL4) emerged vs. control siblings (genotype: w1118;CyO/UAS-dsDop1R2). Total flies assessed, n = 397. (B) Knockdown of Dop1R2 using a third larval salivary gland driver line (i.e., P{GawB}c729-GAL4; FBst0006983, which also targets follicle cells, accessory glands, testis sheath, cyst cells) also results in lethality. At 29 °C, 27.9 % of corresponding dsDop1R2 flies (genotype: w1118;UAS-dsDop1R2/P{GawB}c729-GAL4) emerged vs. adult controls (genotype: w1118;UAS-dsDop1R2, generated in parallel control cross). Total flies assessed, n = 211. In both (A) and (B) the expected dsDop1R2 progeny is 100 % of controls. (C) dsDop1R2 escapers (genotype: w1118;UAS-dsDop1R2/P{GawB} c729-GAL4) display cuticle and wing abnormalities (indicated by arrows in the left and center panels), or fail to fully eclose from pupal case (right panel). (PDF 824 kb) [file 12861_2016_115_MOESM9_ESM.pdf]

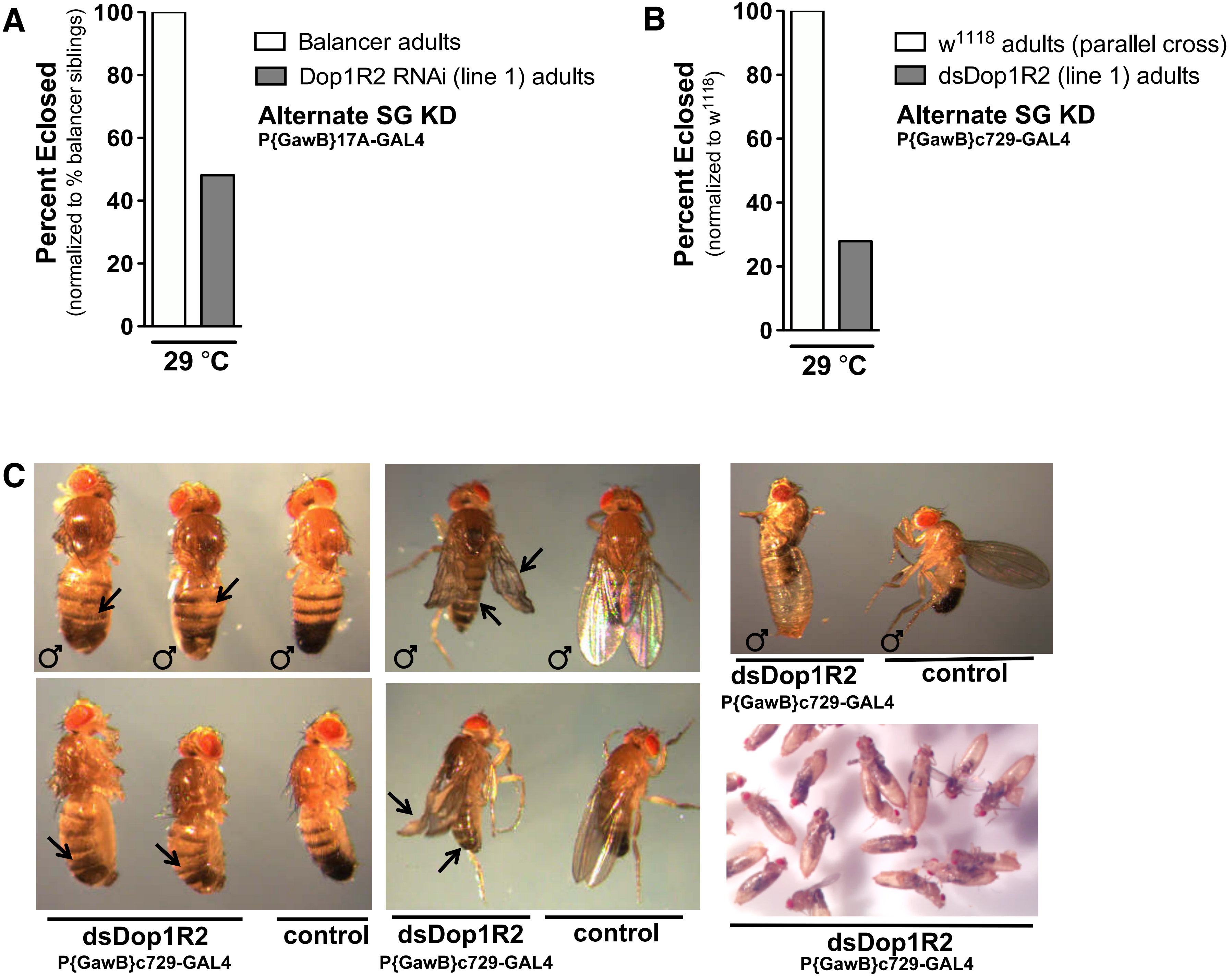
A
Percent Eclosed
(normalized to % balancer siblings)
100
80
60
40
20
0
Balancer adults
Dop1R2 RNAi (line 1) adults
Alternate SG KD
P{GawB}17A-GAL4
29 °C
B
Percent Eclosed
(normalized to w1118)
w1118 adults (parallel cross)
dsDop1R2 (line 1) adults
Alternate SG KD
P{GawB}c729-GAL4
29 °C
C
dsDop1R2
P{GawB}c729-GAL4
control
dsDop1R2
P{GawB}c729-GAL4
control
dsDop1R2
P{GawB}c729-GAL4
control
dsDop1R2
P{GawB}c729-GAL4
